# Synergizing Polysulfide Trapping and Fast Ion Kinetics Enabled by Poly(Acrylamide‐co‐Lithium Acrylate) Aqueous Binder for High‐Sulfur‐Loading Lithium–Sulfur Batteries

**DOI:** 10.1002/smll.73929

**Published:** 2026-05-25

**Authors:** Seongbae Park, Moongil Bang, Seungmok Kang, Seongin Lee, Young‐Jun Kim

**Affiliations:** ^1^ SKKU Advanced Institute of Nano Technology (SAINT) Sungkyunkwan University Suwon Republic of Korea; ^2^ Department of Nano Science and Technology Sungkyunkwan University Suwon Republic of Korea; ^3^ SKKU Institute of Energy Science and Technology (SIEST) Sungkyunkwan University Suwon Republic of Korea

**Keywords:** aqueous binder, high sulfur content, lithium–sulfur batteries, poly(acrylamide‐co‐lithium acrylate), polysulfide adsorption

## Abstract

Although lithium–sulfur (Li–S) batteries offer high theoretical energy density, their practical implementation is impeded by the polysulfide shuttle effect and mechanical disintegration of sulfur cathodes, particularly under high‐sulfur‐loading conditions. To overcome these limitations, a multifunctional aqueous binder, poly(acrylamide‐co‐lithium acrylate) (AmLA), is proposed to synergistically enhance electrode integrity and suppress lithium polysulfide (LiPS) migration. The copolymer architecture integrates polar amide and lithium carboxylate groups, which establish a robust hydrogen‐bonding network for mechanical stability while simultaneously providing strong chemical anchoring sites for LiPS confinement. In situ UV‐visible spectroscopy corroborates the significant retardation of LiPS dissolution, confirming the suppression of shuttle reactions. Consequently, AmLA‐based sulfur cathodes exhibit superior electrochemical kinetics, delivering a 3.4‐fold enhancement in rate capability at 2 C compared to the polyvinylidene fluoride binder. With a high sulfur content of 75 wt% and an areal loading of 3.2 mg cm^−2^, the cathode retains a high reversible capacity of 777 mAh g^−1^ after 200 cycles, accompanied by reduced polarization. Furthermore, stable cycling is preserved even at a high sulfur loading of 6 mg cm^−2^ (7 mAh cm^−2^). Thus, AmLA offers a viable strategy for resolving the chemical and mechanical bottlenecks of high‐energy‐density Li–S batteries.

## Graphical abstract

A water‐soluble AmLA binder anchors lithium polysulfides and reinforces sulfur cathodes by combining amide and lithium acrylate functional groups. The dual‐function network retards polysulfide dissolution in operando and preserves electrode integrity under large volume changes. This design delivers fast kinetics and durable cycling at practical sulfur fractions and areal loadings.

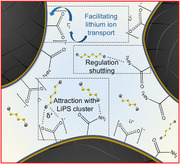

## 1 Introduction

The escalating demand for high‐energy storage systems, ranging from portable electronics to electric vehicles, necessitates next‐generation batteries capable of surpassing the energy density limits of conventional lithium‐ion batteries [1, 2] Lithium–sulfur (Li─S) batteries have emerged as one of the most promising candidates owing to their exceptional theoretical energy density (2600 Wh kg^−1^) and the natural abundance, low cost, and environmental friendliness of sulfur [3, 4, 5]. However, their commercial viability is severely impeded by intrinsic challenges, including the electrically insulating nature of sulfur and its discharge products [6], substantial volumetric expansion of the cathode during cycling [7], and the instability of the lithium metal anode [8]. Most critically, the “polysulfide shuttle effect”—wherein soluble lithium polysulfide (LiPS) intermediates migrate between electrodes—causes continuous loss of active materials and rapid capacity fading, consequently degrading the electrochemical performance and cycle life [9, 10]. Recent efforts to address this issue have included physical confinement, chemisorption, catalytic conversion, and spatial regulation strategies for restricting LiPS migration and accelerating sulfur redox kinetics [11, 12, 13].

To surmount these interrelated obstacles, a holistic strategy is required, with the cathode binder playing a pivotal role in preserving structural integrity and mitigating LiPS migration [14, 15]. The commonly used binder, polyvinylidene fluoride (PVDF), generally fails to accommodate the significant volume fluctuations of sulfur due to its lack of mechanical robustness, despite its chemical stability [16]. Furthermore, the non‐polar nature of PVDF results in weak interactions with polar LiPS species, rendering it ineffective in suppressing the shuttle phenomenon [17]. These limitations have spurred the development of water‐processable polymer binders incorporating polar functional groups to simultaneously enhance electrode integrity and polysulfide regulation [18, 19].

Various polar moieties, such as amine [20], carboxylic acid [21], lithium carboxylate [22], hydroxyl [23], and amide groups [24], have been integrated into polymer backbones to boost polarity and binding affinity toward LiPS. These groups effectively immobilize LiPS through strong chemical interactions, specifically Li–O, Li–N, and S–O bonds, significantly restricting their diffusion [25, 26]. Additionally, the robust interfacial adhesion established between the active material, conductive agents, and current collector contributes to the mechanical durability of the electrode [27].

Recent literature has particularly emphasized the efficacy of polyacrylamide (PAM) [28, 29] and lithium polyacrylate (LiPAA) [30, 31], as their amide and lithium carboxylate groups possess electronegative oxygen and nitrogen atoms capable of forming strong coordinate bonds with LiPS [21, 24]. Moreover, lithium acrylate moieties have been reported to improve electrode‐level Li^+^ transport behavior, leading to enhanced redox kinetics and rate capability [32].

In this study, we propose a multifunctional aqueous binder, poly(acrylamide‐co‐lithium acrylate) (AmLA), designed by chemically integrating the complementary functionalities of acrylamide and lithium acrylate into a unified copolymeric framework. Unlike a simple physical blend of PAM and LiPAA, this structural strategy enables precise modulation of the monomeric ratio within a single polymer backbone, facilitating a synergistic effect to enhance the electrochemical performance of sulfur cathodes. By optimizing the composition through controlled hydrolysis, AmLA ensures strong adhesion to electrode components and efficient LiPS confinement via chemical anchoring, while simultaneously improving electrode‐level ion‐transport behavior in the composite sulfur cathode. Comparative analysis with conventional binders demonstrates that the AmLA binder significantly improves the electrochemical and mechanical performance of high sulfur loading cathodes. This work offers a viable and practical solution to the critical limitations of Li–S battery systems, paving the way for high‐energy‐density applications.

## 2 Results and Discussion

### 2.1 Synthesis and Characterization of AmLA

**FIGURE 1:**
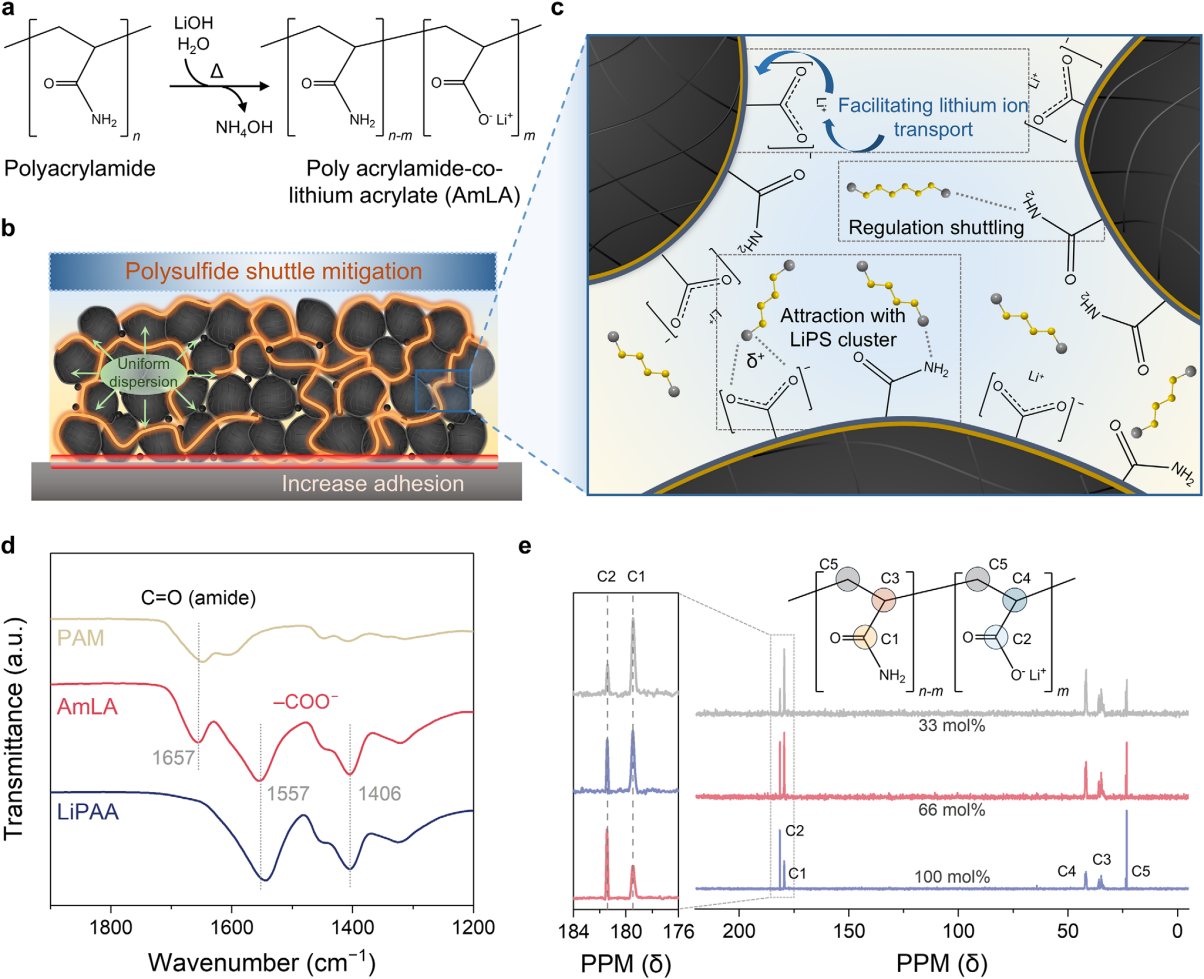
Synthesis strategy and structural characterization of the multifunctional AmLA binder. (a) Reaction scheme for the alkaline hydrolysis of polyacrylamide (PAM) and a schematic illustration of the electrode configuration. (b) Conceptual diagram at the electrode level demonstrating the beneficial effects of AmLA on cathode slurry processing. Key advantages include the uniform dispersion of electrode components, improved adhesion, and the mitigation of the polysulfide shuttle effect. (c) Molecular‐level schematic depicting the proposed synergistic roles of amide and lithium carboxylate groups in enhancing affinity for LiPS and facilitating local Li^+^ transport. (d) Fourier‐transform infrared (FTIR) spectra of pristine PAM, the lithium polyacrylate (LiPAA) reference, and the synthesized AmLA. These spectra confirm the successful incorporation of lithium carboxylate groups into the polymer backbone. (e) ^13^C NMR spectra of the carbonyl region for AmLA synthesized with varying initial LiOH concentrations, demonstrating the tunable composition of acrylamide and lithium acrylate units.

The AmLA binder was synthesized via the controlled alkaline hydrolysis of PAM using lithium hydroxide (LiOH) (Figure 1a). Under these strongly alkaline conditions, hydroxide ions partially hydrolyzed the amide groups, directly yielding lithium carboxylate moieties rather than free carboxylic acids. The reaction was conducted at an elevated temperature (80°C) to provide sufficient thermal energy to overcome the activation energy barrier for hydrolysis [33].

The resulting copolymer architectures, featuring both amide and lithium carboxylate functionalities, markedly influenced the rheological properties of the binder solution. Compared with pristine PAM and fully converted LiPAA, the optimized AmLA solution exhibits higher viscosity at low shear rates while maintaining pronounced shear‐thinning behavior, which collectively signifies enhanced slurry stability and coatability during electrode processing (Figure S1). This rheological enhancement originates from the reinforced interpolymer networks established through dual‐functional interactions, specifically the combination of hydrogen bonding among amide groups and ionic associations between lithium carboxylate units. Such a dual‐interaction mechanism is responsible for the significant viscosity increase observed at intermediate conversion ratios. These rheological characteristics provide robust experimental evidence for the improved slurry processability schematically illustrated in Figure 1b, particularly for cathode formulations with high content ratio of conductive carbon. Furthermore, at the molecular level, these functional groups are proposed to provide affinity for LiPS and facilitate local Li^+^ transport, consequently contributing to effective polysulfide shuttle mitigation (Figure 1c).

The chemical structure of the synthesized AmLA was verified using Fourier transform infrared (FTIR) spectroscopy (Figure 1d). The spectrum of PAM exhibits the characteristic C═O stretching vibration of amide groups at 1650 cm^−1^, whereas LiPAA displays lithium carboxylate peaks at 1557 and 1406 cm^−1^ [34]. The emergence of these carboxylate bands in the AmLA spectrum, alongside the retained amide peak, confirms the successful partial conversion of amide groups into lithium carboxylates. The degree of hydrolysis was precisely tuned by adjusting the molar feed ratio of LiOH to amide units.

To quantify the relative composition of acrylamide and lithium acrylate moieties, ^13^C nuclear magnetic resonance (NMR) spectroscopy was performed on AmLA samples synthesized with varying LiOH‐to‐amide ratios (Figure 1e). Carbon signals corresponding to the carbonyl carbons of the amide and lithium carboxylate groups were identified at approximately 179.5 and 181.5 ppm, respectively [35]. As the LiOH‐to‐amide ratio increased, the intensity of the lithium carboxylate signal rose while the amide signal concurrently diminished. This trend confirms that the chemical composition and degree of conversion can be systematically controlled via the LiOH feed concentration.

### 2.2 Optimization of the Conversion Ratio

**FIGURE 2:**
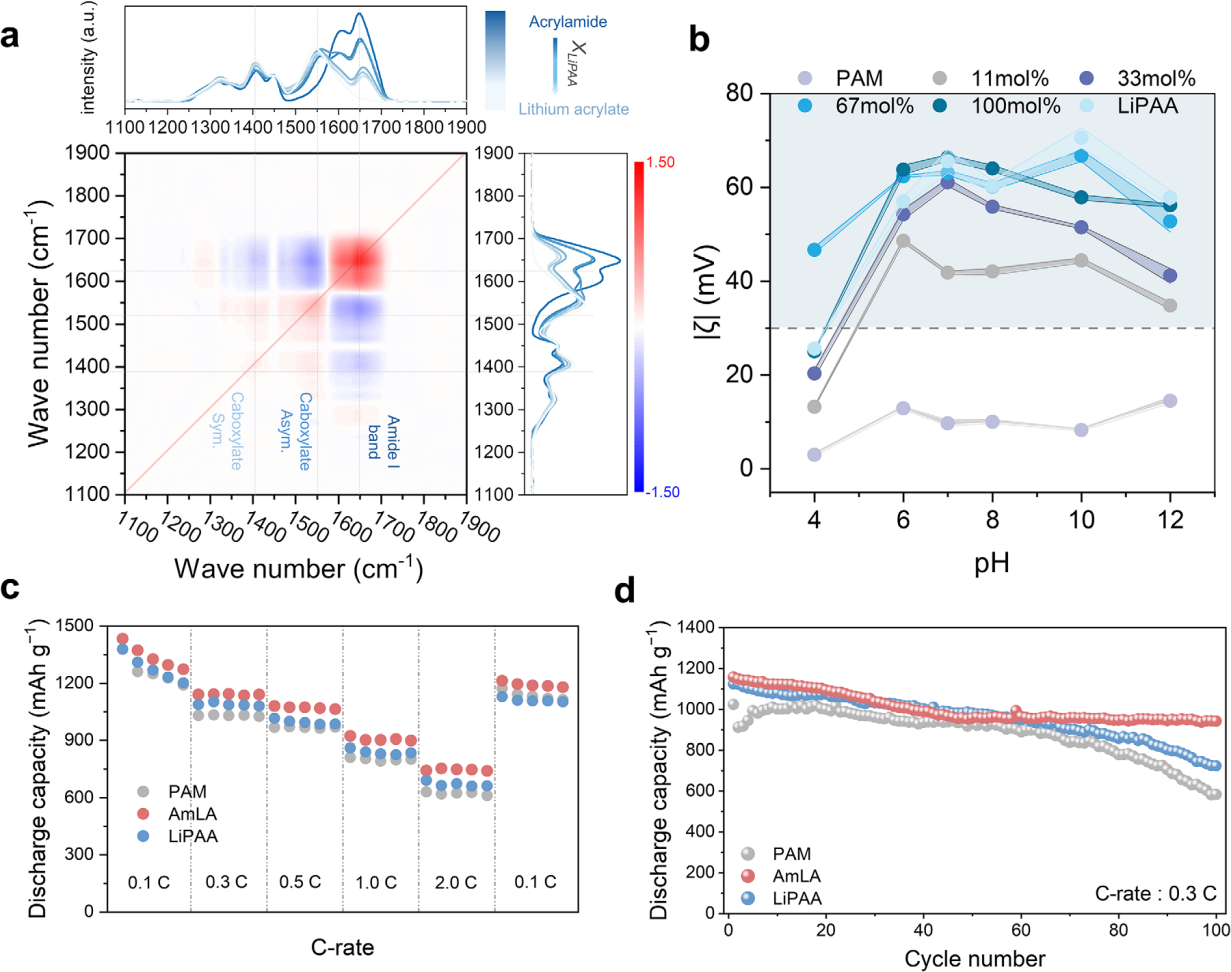
Optimization of the AmLA binder composition and its impact on electrochemical performance. (a) Synchronous 2D correlation FTIR spectra of AmLA binders synthesized with varying hydrolysis ratios, visualizing the structural transition from amide to lithium carboxylate groups. (b) Zeta potential measurements as a function of pH for PAM, AmLA with varying degrees of hydrolysis, and the fully converted LiPAA reference, highlighting the tunable surface charge properties. (c) Rate capability of sulfur cathodes utilizing different binders, evaluated at C‐rates ranging from 0.1 to 2 C with a fixed sulfur loading of 3.2 mg cm^−2^. (d) Cycling performance of the corresponding cathodes tested at a constant rate of 0.3 C, confirming the superior stability of the optimized AmLA formulation (33 mol%).

To optimize the binder functionality, the impact of the AmLA conversion ratio on the binding properties and electrochemical performance was systematically investigated (Figure 2). In the FTIR spectra, the characteristic amide band at ∼1650 cm^−1^ gradually decreased, while the carboxylate bands assigned to –COO^−^Li^+^ at ∼1557 and 1406 cm^−1^ intensified with higher conversion ratios. To elucidate the specific structural evolution during hydrolysis, 2D correlation Fourier transform infrared (2D‐COS‐FTIR) spectroscopy was utilized. This analysis demonstrated a positive correlation between the carboxylate peaks (1557/1406 cm^−1^) and a negative correlation with the amide band (1650 cm^−1^) [36]. These synchronous spectral changes indicate that the transformation of amide groups into lithium carboxylates proceeds proportionally with increasing LiOH content (Figure 2a). This observation aligns with the raw FTIR spectra of the partially converted polymers and the corresponding band assignments detailed in Figure S2.

The colloidal stability of the binder solution was subsequently assessed via zeta potential measurements (Figure 2b). While pristine PAM exhibited only a weak surface charge, AmLA displayed progressively more negative values as the conversion ratio increased. It is noteworthy that the 33 mol% AmLA maintained a zeta potential below −30 mV under near‐neutral conditions. This value is generally recognized as the threshold for ensuring colloidal stability through electrostatic repulsion [37]. Although the fully converted LiPAA also exhibited a strong negative potential, it lacks the amide functionalities necessary to support the hydrogen‐bonding networks required for mechanical resilience. These surface charge characteristics correspond well with the zeta potential distributions presented in Figure S3.

The rheological behavior further reflected this structural balance, with the solution viscosity reaching a maximum at 33 mol% conversion before declining at higher ratios (Figure S1). Corresponding morphological variations were observed in scanning electron microscopy (SEM) images of electrodes prepared with 33, 67, and 100 mol% AmLA (Figure S4), highlighting the structural differences associated with the degree of conversion. Among the investigated compositions, the 33 mol% AmLA‐based electrode exhibits the most compact and cohesive morphology, characterized by a significant reduction in structural defects and surface cracks. This optimized microstructure is a direct consequence of the balanced coexistence between residual amide groups and lithium carboxylate units. Specifically, the sufficient density of amide groups facilitates a robust, binder‐mediated cohesive network via interpolymer hydrogen bonding, which effectively anchors the electrode components. In contrast, electrodes with higher conversion ratios display less consolidated structures with increased porosity and visible defects, likely due to the diminished amide content weakening the hydrogen‐bonding‐driven cohesion. Such enhanced morphological integrity is instrumental in accommodating the large volume fluctuations inherent to sulfur conversion reactions, thereby preserving intimate interparticle contact and ensuring long‐term structural stability during repeated cycling. Electrochemical evaluation confirmed that the intermediate conversion ratio was indeed the most favorable. In the rate capability and cycling tests (Figures 2c,d), the 33 mol% AmLA‐based cathode significantly outperformed both PAM and LiPAA, a result consistent with the detailed cyclic voltammetry (CV) curves and Randles–Sevcik analysis (Figure S5). Adhesion strength tests further revealed that the partially converted AmLA electrodes exhibited higher peel resistance than both fully converted LiPAA and conventional binders, as shown in the representative peel curves and averaged values (Figures S6 and S7). Furthermore, the performance of AmLA prepared via NaOH hydrolysis was inferior to that of LiOH‐derived AmLA, underscoring the critical role of the counter‐cation in facilitating ion transport (Figure S8). Extended cycling and rate capability for the series of binders further corroborated these results (Figure S9).

Consequently, a 33 mol% AmLA formulation is identified as the optimal configuration, delivering a superior combination of dispersion stability, mechanical integrity, and electrochemical performance. This optimized balance is attributed to the synergistic coexistence of lithium carboxylate (–COO^−^Li^+^) groups, which enhance polysulfide affinity and electrode‐level ion transport, and residual amide groups, which reinforce interparticle adhesion and electrode robustness.

### 2.3 Mechanical Strength and Structural Morphology

**FIGURE 3:**
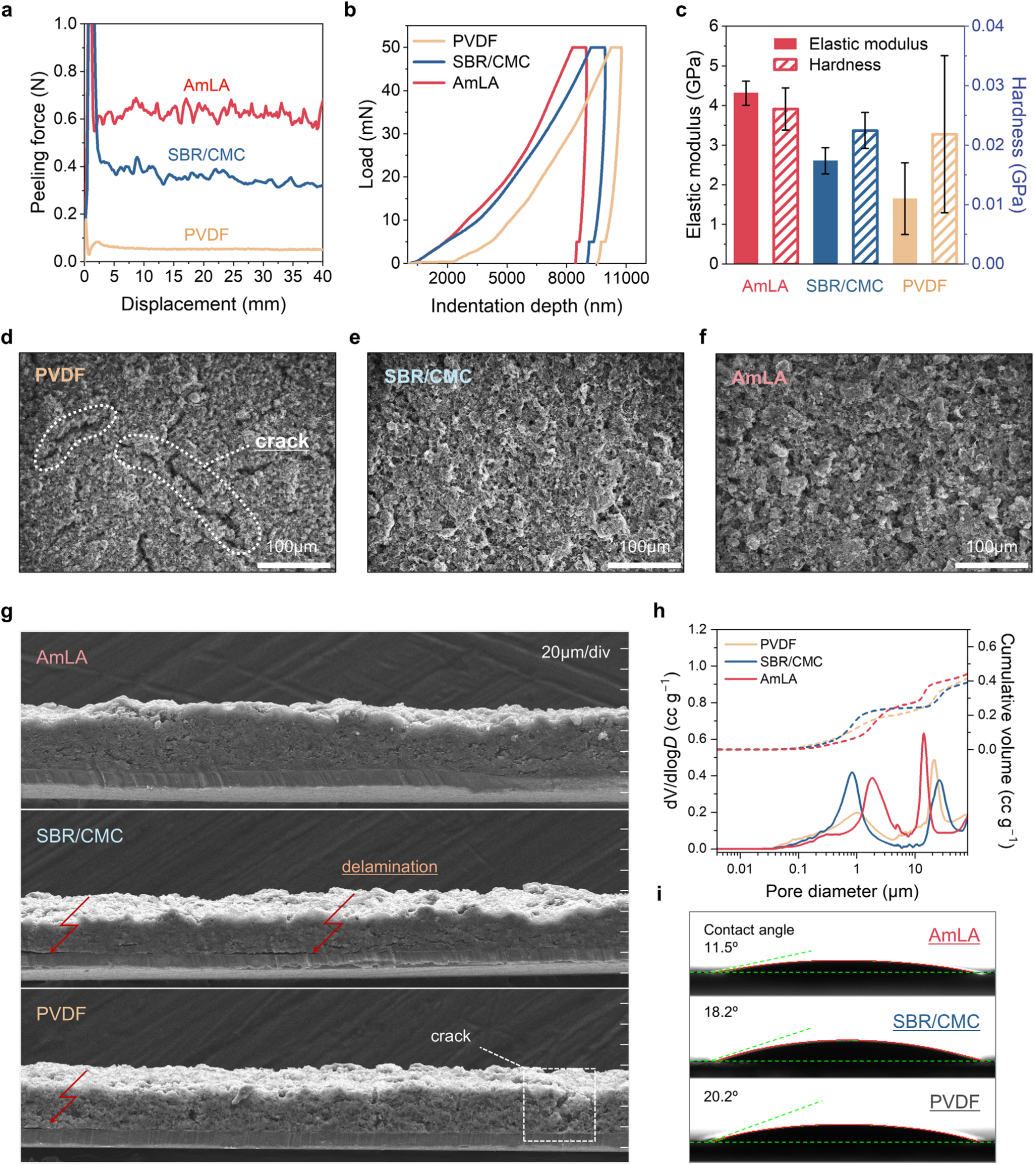
Comprehensive mechanical robustness and structural characterization of sulfur cathodes employing different binder systems. (a) Comparative peeling force–displacement profiles obtained from T‐peel tests for PVDF, SBR/CMC, and AmLA‐based electrodes. (b) Load‐displacement hysteresis loops derived from nanoindentation measurements and (c) the corresponding quantitative comparison of elastic modulus and hardness values, confirming the superior resistance of AmLA to plastic deformation. (d–f) SEM images showing the surface morphologies of electrodes prepared with (d) PVDF, (e) SBR/CMC, and (f) AmLA binders. (g) Corresponding cross‐sectional micrographs of the electrodes. These images highlight the cohesive and crack‐free architecture of the AmLA electrode, which stands in sharp contrast to the extensive fracturing and delamination observed in the PVDF and SBR/CMC samples. (h) Pore size distribution and cumulative pore volume, revealing that AmLA establishes a more uniform and well‐connected porous network. (i) Contact angle measurements demonstrating the superior electrolyte wettability of the AmLA surface relative to the control binders. This enhanced hydrophilicity facilitates rapid electrolyte infiltration and efficient ion flux.

The mechanical integrity of the cathode is a prerequisite for stable cycling, particularly given the substantial volume expansion of sulfur during its lithiation to Li_2_S [38]. Consequently, the mechanical robustness and structural stability of the AmLA binder were rigorously evaluated against commercial benchmarks, specifically styrene–butadiene rubber/carboxymethyl cellulose (SBR/CMC) and PVDF (Figure 3). The interfacial adhesion strength was initially quantified via T‐peel tests (Figure 3a). The AmLA‐based electrode demonstrated a superior peeling force of 0.68 N, significantly outperforming SBR/CMC (0.40 N) and PVDF (0.05 N). This enhancement is attributed to the abundant polar functional groups (–COO^−^Li^+^ and –CONH_2_) along the AmLA backbone. These moieties establish a robust hydrogen‐bonding network and strong coordination with the current collector and active materials. In contrast, PVDF relies predominantly on weak van der Waals forces, resulting in insufficient binding strength and susceptibility to delamination. These quantitative results are visually supported by optical images of the peeled electrodes presented in Figure S10.

To further probe the micromechanical properties at the particle–binder interface, nanoindentation measurements were conducted (Figure 3b,c). The AmLA electrode exhibited the minimum indentation depth (8451 nm at a 50 mN load) alongside the highest elastic modulus (4.3 GPa) and hardness (0.026 GPa) among the tested binders. These metrics indicate exceptional resistance to external stress and plastic deformation, suggesting that the AmLA binder effectively accommodates the internal stress generated during the sulfur volume expansion. Additionally, the favorable rheological behavior of AmLA relative to SBR/CMC and PVDF, as elucidated by the viscosity–shear profiles (Figure S11), underscores its superior processability and ability to form uniform electrode films without segregation.

The structural benefits of AmLA were further corroborated by SEM. While PVDF‐based electrodes suffered from extensive surface cracking and SBR/CMC electrodes displayed aggregation with partial delamination, the AmLA electrode maintained a uniform and compact morphology devoid of obvious defects, as observed in both surface and cross‐sectional views (Figure 3d–f). Figure S12 provides further insight into the pore distribution. In contrast to the severe cracking observed in PVDF and the limited porosity of SBR/CMC, AmLA preserved a homogeneous porous architecture favorable for efficient ion transport.

Mercury intrusion porosimetry analysis further confirmed that AmLA establishes a well‐optimized porous network (Figure 3h). This structure provides abundant pathways for ion transport while simultaneously preventing the excessive aggregation of conductive additives. Complementing the structural analysis, contact angle measurements demonstrated the superior electrolyte wettability of the AmLA binder. The AmLA surface exhibited a contact angle of 11.5°, which is notably lower than that of SBR/CMC (18.2°) and PVDF (20.2°) (Figure 3i). This enhanced hydrophilicity facilitates rapid electrolyte infiltration, leading to the effective utilization of the active material. Collectively, these improvements in interfacial adhesion, mechanical robustness, pore architecture, and wettability empower the AmLA binder to maintain a stable electrode configuration. This structural and chemical integrity serves as the foundation for the enhanced electrochemical performance observed in sulfur cathodes.

### 2.4 Polysulfide Adsorption Capability

**FIGURE 4:**
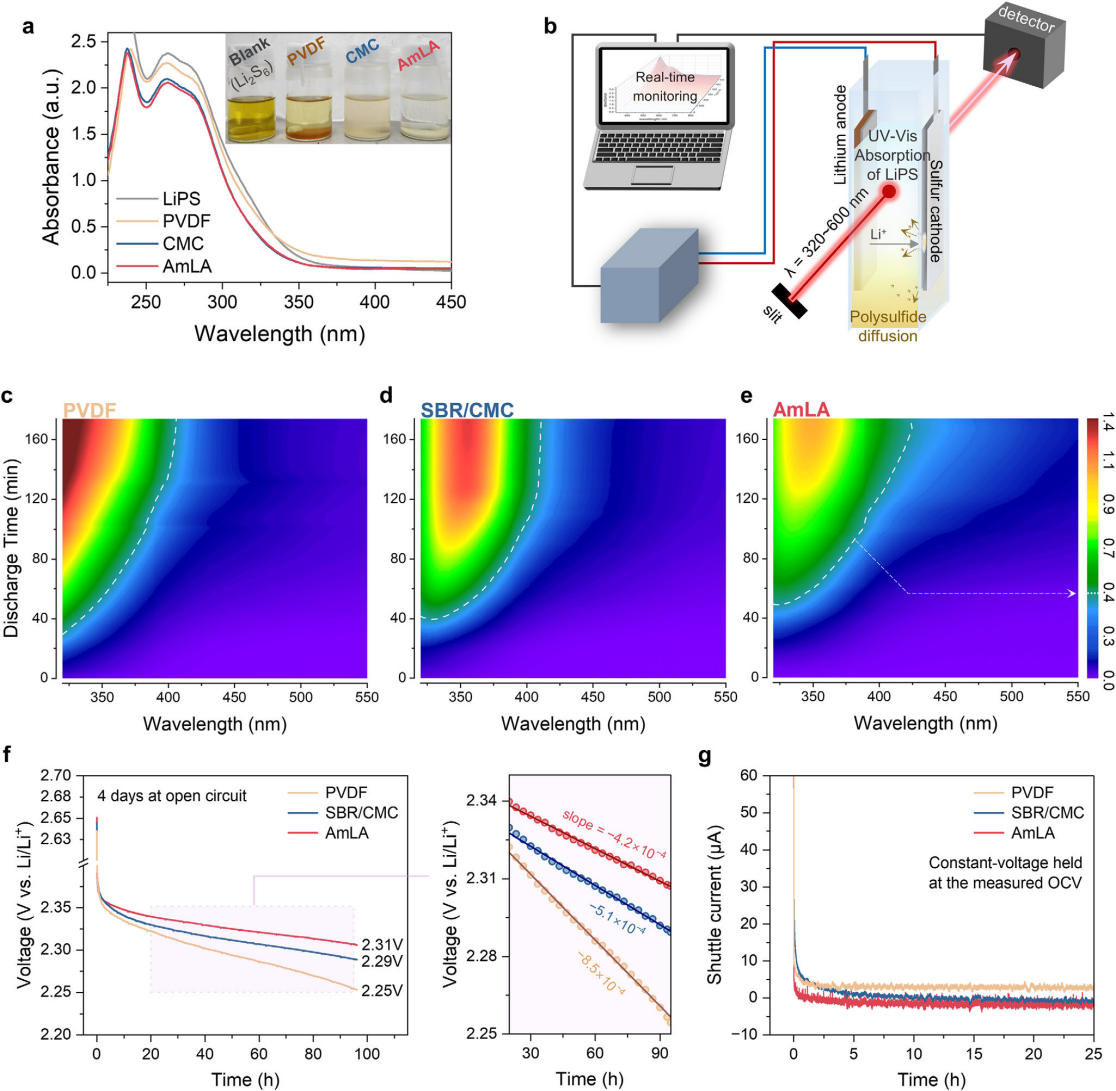
Evaluation of polysulfide regulation capability and shuttle suppression efficacy. (a) Comparative UV–vis absorption spectra of Li_2_S_6_ solutions after exposure to PVDF, CMC, and AmLA powders for 24 h. The inset provides digital photographs showing the varying degrees of decolorization, visually confirming the strong chemical affinity of AmLA for polysulfides. (b) Schematic illustration of the in situ UV–vis spectroscopy setup employed to monitor the real‐time evolution of polysulfides during the discharge process. (c–e) Time‐resolved contour maps of in situ UV–vis spectra obtained from sulfur cathodes utilizing (c) PVDF, (d) SBR/CMC, and (e) AmLA binders. The significantly reduced absorbance intensity and delayed signal onset in the AmLA‐based cell demonstrate the effective confinement of soluble intermediates within the cathode. (f) Open‐circuit voltage profiles of Li–S cells monitored over a resting period of 4 days. The AmLA cell exhibits superior voltage retention, indicating the successful mitigation of self‐discharge behavior. (g) Shuttle current leakage measurements recorded under potentiostatic conditions. These results confirm that the AmLA electrode exhibits the most effective suppression of the polysulfide shuttle effect, minimizing parasitic redox reactions.

The effective mitigation of the soluble LiPS shuttle effect is paramount for enhancing the reversibility and cycling stability of sulfur cathodes. To comprehensively evaluate the adsorption capability and shuttle suppression efficacy of the AmLA binders, a multi‐faceted approach involving ex situ and in situ UV‐visible (UV–vis) spectroscopic analyses was employed, complemented by electrochemical self‐discharge and shuttle current measurements (Figure 4).

In the ex situ adsorption experiment, the stoichiometric Li_2_S_6_ solution was prepared, initially exhibiting a characteristic yellow hue and a strong absorption peak at 265 nm [39]. After 24 h of exposure to the binder powders, the solution treated with AmLA became nearly transparent, whereas those containing PVDF and CMC retained appreciable coloration and absorbance (Figure 4a). This distinct visual and spectral transformation indicates that the AmLA binder possesses a superior affinity for LiPS, efficiently sequestering them from the electrolyte. Figure S13 presents the corresponding optical images and UV–vis spectra, further highlighting the enhanced adsorption performance of AmLA compared to both PVDF and LiPAA. This robust adsorption capability is attributed to the abundant polar amide (–CONH_2_) and lithium acrylate (–COO^−^Li^+^) functionalities, which electrostatically interact with soluble LiPS species through stable Li–O and Li–N coordination bonds.

To probe the polysulfide regulation mechanism under dynamic electrochemical conditions, in situ UV–vis spectroscopy was performed using a customized Li–S cell assembled with a quartz cuvette (Figure 4b). The detailed cell configuration and optical images capturing the polysulfide dissolution process are provided in Figure S14. Time‐resolved contour maps recorded during galvanostatic discharge reveal distinct differences in LiPS diffusion among the binders (Figure 4c–e). The PVDF electrode, lacking polar anchoring sites, exhibited the most intense absorbance signals immediately upon discharge, while SBR/CMC showed moderate suppression. By contrast, the AmLA electrode displayed the lowest absorbance intensity and a significantly delayed onset of polysulfide detection. Specifically, the detection of dissolved LiPS, defined at an absorbance threshold of 0.4, occurred much later for the AmLA electrode compared to its counterparts. Furthermore, the relative absorbance level of 0.9 (yellow region) was reached at approximately 70 min for PVDF and 80 min for SBR/CMC, whereas the AmLA electrode successfully delayed this saturation point to 120 min. These results demonstrate that AmLA effectively retards the outward diffusion of LiPS during the S_8_ → Li_2_S_4_ transition, consequently minimizing their accumulation in the bulk electrolyte. Such confinement facilitates a more efficient conversion in the subsequent plateau (Li_2_S_4_ → Li_2_S_2_/Li_2_S), a process that dominates the overall discharge capacity.

Beyond spectroscopic evidence, the functional impact of polysulfide regulation was verified through self‐discharge and shuttle current tests. During open‐circuit storage for four days, cells employing AmLA retained higher and more stable potentials than those using PVDF or SBR/CMC (Figure 4f), highlighting the reduced parasitic loss of active sulfur species [40]. Moreover, under constant‐voltage conditions, the AmLA electrode exhibited the lowest shuttle current throughout the measurement window (Figure 4g), whereas PVDF and SBR/CMC electrodes showed larger, sustained leakage currents. This significant suppression of the shuttle current confirms that the AmLA binder effectively blocks the continuous redox cycling of dissolved polysulfides, which acts as a primary degradation pathway in Li–S cells.

Consequently, the diffusion of LiPS is effectively hindered by the AmLA electrode owing to the strong chemical affinity between its polar functional groups and the polysulfide species. This suppression alleviates continuous shuttle‐driven side reactions and preserves soluble intermediates within the cathode region. By confining LiPS during the early stages of discharge, AmLA promotes a more efficient conversion toward solid Li_2_S_2_/Li_2_S, a phenomenon further examined in the subsequent analyses of nucleation and ionic transport.

### 2.5 Li_2_S Nucleation and Deposition Behavior

**FIGURE 5:**
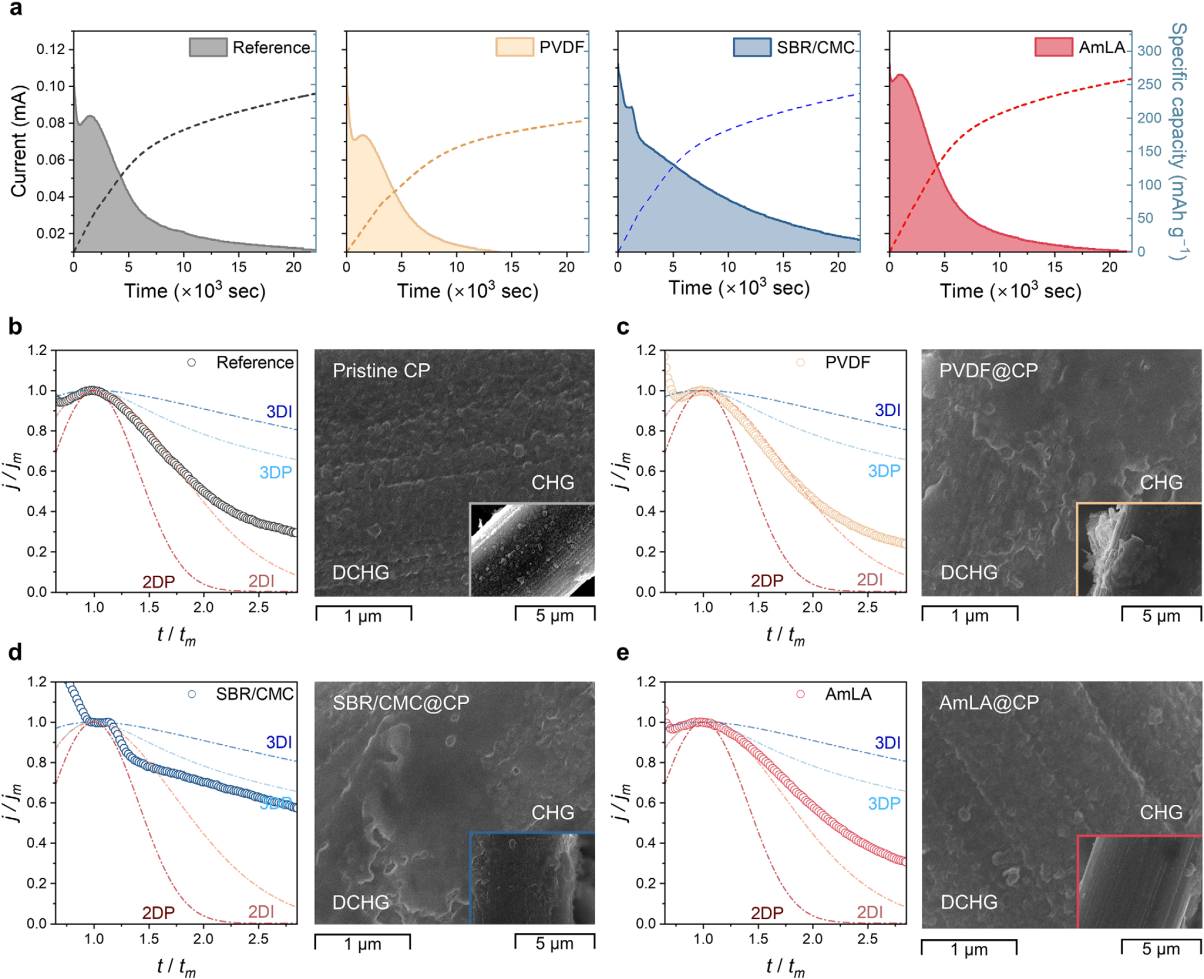
Elucidation of Li_2_S nucleation kinetics and deposition/dissolution reversibility on binder‐modified carbon paper (CP) substrates. (a) Potentiostatic discharge transients of CP electrodes modified with PVDF, SBR/CMC, and AmLA binders recorded in a Li_2_S_8_ catholyte. (b–e) Dimensionless current–time plots compared with theoretical nucleation models for (b) bare CP as a reference, (c) PVDF, (d) SBR/CMC, and (e) AmLA. The corresponding SEM images visualize the morphology of Li_2_S deposits after full discharge (DCHG, main panels) and subsequent charging (CHG, insets). The PVDF and SBR/CMC electrodes exhibit kinetics aligning with 2D growth models, where SEM images reveal the formation of dense, film‐like layers that tend to passivate the surface. Crucially, the charging insets for these binders show significant residual deposits, indicating incomplete dissolution and poor reversibility. In distinct contrast, the AmLA binder promotes 3D progressive (3DP) nucleation, evidenced by the formation of discrete, granular Li_2_S particles. This 3D morphology prevents premature passivation and facilitates complete dissolution upon charging, as confirmed by the pristine electrode surface observed in the AmLA inset.

To elucidate the precipitation kinetics of Li_2_S and the deposition characteristics of LiPS species, potentiostatic nucleation tests were conducted using Li_2_S_8_ catholyte on carbon paper (CP) electrodes. The CP electrodes served as the substrate, including bare CP as a reference and CP impregnated with PVDF, SBR/CMC, or AmLA. Following the injection of 40 µL of Li_2_S_8_ catholyte, the current–time transients were recorded under potentiostatic discharge (Figure 5a) [41]. The PVDF‐CP exhibited a current response lower than that of bare CP, which can be attributed to its non‐polar nature and weak interaction with LiPS. In contrast, SBR/CMC‐CP and AmLA‐CP demonstrated significantly higher peak currents and capacity integration. To further substantiate this enhanced kinetic behavior, cyclic voltammetry (CV) was performed on symmetric cells using identical electrodes (Figure S15). While the PVDF and bare CP cells showed negligible redox activity, the AmLA‐based cell exhibited the highest current density with well‐defined peaks. This result serves as direct evidence that the AmLA binder possesses superior electrocatalytic activity, effectively accelerating the polysulfide redox conversion. Consequently, the polar functional groups inherent in the AmLA binder effectively lower the nucleation barrier and facilitate the precipitation of Li_2_S.

The deposition mechanism was further analyzed by normalizing the data into dimensionless current–time transients and comparing them with classical nucleation growth models, specifically the 2D thin‐film nucleation (Bewick–Fleischmann–Thirsk) and 3D nucleation (Scharifker–Hills) models (Figure S16) [42, 43]. The transient curves for PVDF and SBR/CMC electrodes were primarily described by 2D instantaneous (2DI) or progressive (2DP) nucleation regimes. In distinct contrast, the AmLA electrode exhibited kinetic behavior characteristic of a mixed regime involving both 2DP and 3D progressive (3DP) nucleation. These kinetic assignments align well with ex situ SEM observations following potentiostatic discharge (Figure 5b–e). The PVDF and SBR/CMC binders induced the formation of dense, film‐like Li_2_S layers that tend to passivate the conductive surface, hindering further reactions. On the other hand, AmLA promoted the growth of 3D, particle‐type Li_2_S deposits, which preserve the active surface area and prevent premature electrode passivation, allowing for deeper discharge capacity.

Upon subsequent charging, SEM imaging revealed a distinct contrast in reversibility (Figure 5b–e, inset). The Li_2_S deposits formed on the AmLA‐modified CP electrode were effectively decomposed, and the electrode surface was largely restored to its pristine state. This demonstrates highly reversible deposition–dissolution behavior. In comparison, the PVDF and SBR/CMC electrodes retained residual film‐like deposits, highlighting their limited reversibility and the consequent accumulation of dead sulfur species. These results indicate that the AmLA binder regulates both the nucleation and redissolution of LiPS more effectively than the conventional binders, ensuring stable electrochemical activity and preventing the accumulation of irreversible discharge products.

### 2.6 Enhanced Li^+^ Diffusivity and Electrochemical Kinetics

**FIGURE 6:**
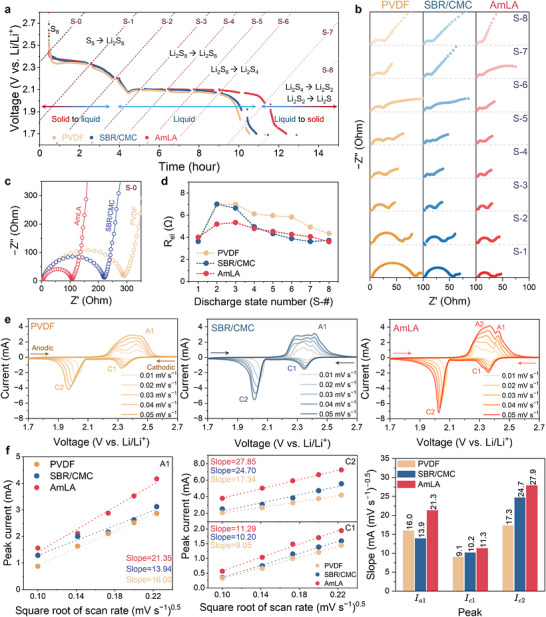
Electrochemical impedance spectroscopy (EIS) and kinetic analysis of Li^+^ transport dynamics. (a) Galvanostatic discharge voltage profiles indicating the specific depth‐of‐discharge (DoD) states (S‐0 to S‐8) selected for operando EIS measurements. (b) Evolution of Nyquist plots for PVDF, SBR/CMC, and AmLA electrodes recorded at the corresponding discharge states. (c) Comparative Nyquist spectra at the initial open‐circuit state (S‐0), highlighting the reduced interfacial resistance of the AmLA electrode. (d) Variation of electrolyte resistance (R_el_) as a function of discharge depth. The AmLA binder minimizes the resistance surge typically associated with polysulfide dissolution and electrolyte viscosity increase. (e) Cyclic voltammograms (CV) acquired at varying scan rates ranging from 0.01 to 0.05 mV s^−1^. (f) Linear fitting of the peak current versus the square root of the scan rate (ν^0.5^) for oxidation (A1) and reduction (C1, C2) peaks. The corresponding slope comparison confirms that AmLA facilitates the fastest Li^+^ diffusion kinetics, particularly accelerating the sluggish liquid–solid conversion process (C2).

The influence of binder architecture on Li^+^ transport kinetics was systematically evaluated using electrochemical impedance spectroscopy (EIS) and CV. The discharge voltage profiles presented in Figure 6a mark the specific depth‐of‐discharge (DoD) states (S‐0 to S‐8) selected for the EIS analysis [44]. The Nyquist plots collected at these various discharge states (Figure 6b) clearly demonstrate that the semicircles of the AmLA electrode are consistently smaller than those of the PVDF and SBR/CMC counterparts, indicating reduced interfacial resistance and accelerated charge transfer kinetics. To ensure a rigorous quantitative analysis, the impedance spectra were fitted using an equivalent circuit model, which is provided along with the individual Nyquist plots for each discharge state in Figure S17. Across the discharge process, the impedance initially decreases from S‐1 to S‐4 as insulating S_8_ is progressively converted into soluble lithium polysulfides, thereby increasing the concentration of electrochemically accessible redox species in the electrolyte. In contrast, the impedance rises again from S‐5 to S‐8, mainly due to the precipitation of insulating Li_2_S_2_/Li_2_S species on the electrode surface during the later liquid–solid and solid–solid conversion stages [45]. Even at the onset of discharge (S‐0, Figure 6c), the AmLA electrode exhibits a lower charge‐transfer resistance (R_ct_), a kinetic advantage that is maintained throughout the entire discharge process. The reduced R_ct_ at S‐0 is attributed to improved interfacial wetting and a larger electrochemically active area enabled by the polar functional groups of AmLA. A quantitative comparison of the electrolyte resistance (R_el_) (Figure 6d) reveals that the performance disparity is most pronounced at states S‐2 and S‐3, the region where the concentration of soluble polysulfides reaches its maximum. Furthermore, both the surface film resistance (R_film_) and R_ct_ values of the AmLA electrode remain consistently lower across all discharge states compared to the other binders. These results suggest that the polar functional groups of AmLA effectively immobilize polysulfides at the electrode interface. This confinement prevents the excessive diffusion of LiPS into the bulk electrolyte, which would otherwise increase the electrolyte viscosity and impede ion transport. In contrast, PVDF shows higher interfacial resistance due to its non‐polar nature, while SBR/CMC demonstrates intermediate behavior.

The Li^+^ diffusion capability was further probed by CV at scan rates ranging from 0.01 to 0.05 mV s^−1^ (Figure 6e). All electrodes exhibit two cathodic peaks, C1 (S_8_ → Li_2_S_4_) and C2 (Li_2_S_4_ → Li_2_S_2_/Li_2_S), together with two anodic peaks, A2 and A1. These anodic peaks represent the stepwise electrochemical oxidation of solid Li_2_S/Li_2_S_2_ back to soluble long‐chain LiPS and eventually to crystalline S_8_, respectively [46]. Notably, in the PVDF and SBR/CMC electrodes, A2 shifts toward A1 as the scan rate increases, resulting in a partial merger of the two peaks due to excessive oxidation polarization of the solid discharge products. In contrast, the AmLA electrode maintains distinct and clearly separated A2 and A1 peaks across all tested scan rates. This behavior reflects significantly reduced polarization and faster redox kinetics for the liquid‐solid phase transitions, further corroborating the efficacy of the AmLA binder in facilitating efficient charge transfer. Among the cathodic processes, the AmLA electrode displays the highest peak currents, particularly for the C2 peak. This peak corresponds to the liquid–solid conversion step, which accounts for nearly 75% of the total discharge capacity. This enhanced peak current implies that AmLA significantly accelerates the kinetics of this critical rate‐determining step. The diffusion coefficient was analyzed according to the Randles–Sevcik equation:

(1)

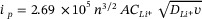

where *i_p_* is the peak current, *n* is the number of transferred electrons, *A* is the electrode area, $C_{Li^{+}}$ is the Li^+^ concentration in the electrolyte, $D_{Li^{+}}$ is the Li^+^ diffusion coefficient, and *v* is the scan rate [47]. The linear relationship between the peak current and the square root of the scan rate is plotted in Figure 6f. The slopes obtained from the linear fitting reflect the effective diffusion coefficient of Li^+^. The AmLA‐based electrode exhibits the steepest slopes across all identified major peaks (A1, C1, and C2), confirming its superior Li^+^ transport characteristics relative to the PVDF and SBR/CMC benchmarks (Table S1). Notably, the slope at C2 is significantly larger for AmLA, reinforcing the conclusion that the binder facilitates rapid Li^+^ migration and efficient phase transformation during the discharge process. Collectively, these results demonstrate that AmLA reduces interfacial resistances and accelerates Li^+^ diffusion throughout the entire electrochemical process. These improvements are attributed to the synergistic effect of the amide and lithium acrylate groups, which simultaneously anchor polysulfides and create ionically conductive pathways. As a result, the AmLA electrode demonstrates enhanced redox kinetics, particularly in the sluggish liquid–solid transition regime, supporting stable and efficient cycling performance in Li–S cells.

### 2.7 Reaction Kinetics and Overpotential Analysis

**FIGURE 7:**
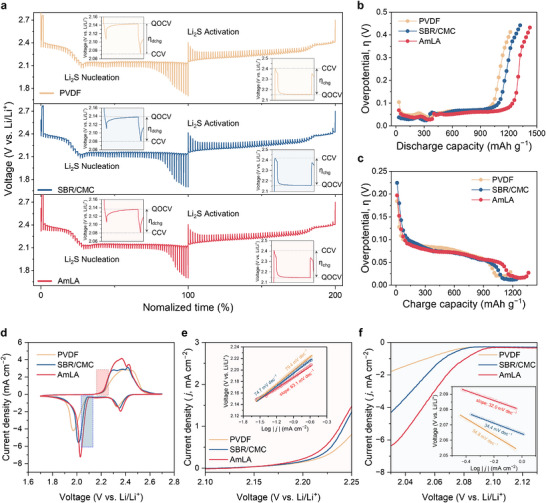
Kinetic analysis of sulfur redox reactions. (a) GITT voltage profiles of Li–S cells employing PVDF, SBR/CMC, and AmLA binders, illustrating the quasi‐equilibrium potentials and polarization behaviors during discharge. (b,c) Evolution of reaction overpotential (η) derived from GITT curves during (b) the discharge process and (c) the charge process. The AmLA electrode exhibits the lowest polarization across the entire capacity range. (d) Tafel plots corresponding to the reduction reaction (Li_2_S_4_ → Li_2_S), fitted to calculate the cathodic Tafel slopes. (e, f) Tafel plots for the oxidation reaction (Li_2_S → Li_2_S_4_) comparing (e) PVDF and SBR/CMC against (f) AmLA, highlighting the reduced anodic slope for the AmLA‐based cell. These kinetic metrics confirm that the AmLA binder effectively lowers the activation energy for both the nucleation of solid Li_2_S and its subsequent oxidation.

The stepwise reduction kinetics of sulfur in Li–S cells were deconstructed using the galvanostatic intermittent titration technique (GITT) and Tafel polarization measurements (Figure 7). In the GITT discharge profiles (Figure 7a), all electrodes delivered nearly identical capacities at the end of the first plateau (S_8_ → Li_2_S_4_). However, the second plateau (Li_2_S_4_ → Li_2_S_2_/Li_2_S), which corresponds to the kinetically sluggish liquid–solid phase transformation, exhibited distinct binder‐dependent behaviors. The AmLA electrode sustained a prolonged plateau with significantly reduced polarization, whereas the PVDF and SBR/CMC counterparts displayed earlier voltage decay. This contrast indicates that the AmLA binder exerts stronger regulation over soluble polysulfides, facilitating their complete conversion.

The evolution of reaction overpotential (η) during the discharge and charge processes further reflects these kinetic differences. As shown in Figure 7b, the AmLA electrode maintains a consistently lower discharge overpotential over the majority of the discharge capacity compared to the PVDF and SBR/CMC electrodes. Notably, the onset of the sharp increase in η—a phenomenon associated with the accumulation of insulating and ionically resistive Li_2_S_2_/Li_2_S—is significantly delayed toward higher discharge capacities in the AmLA‐based system. This characteristic trend indicates that the AmLA binder effectively suppresses electrochemical polarization during the critical sulfur conversion stages. During the subsequent charging process (Figure 7c), the AmLA electrode also exhibits a more stable overpotential evolution, suggesting facilitated oxidation of the solid discharge products and reduced kinetic hindrance during the reverse conversion. These results demonstrate that AmLA significantly mitigates kinetic polarization, particularly in the critical second plateau region where the deposition of insulating Li_2_S typically impedes charge transfer [48].

Tafel analysis provided further quantitative insight into the interfacial kinetics (Figure 7d). The calculated cathodic Tafel slopes were 63.1 mV dec^−1^ for AmLA, 74.7 mV dec^−1^ for SBR/CMC, and 79.4 mV dec^−1^ for PVDF. The lowest slope observed for AmLA confirms accelerated Li_2_S nucleation kinetics. For the anodic branch, AmLA also showed the lowest slope of 32.0 mV dec^−1^, compared to 34.4 mV dec^−1^ for SBR/CMC and 54.8 mV dec^−1^ for PVDF (Figure 7e,f). Furthermore, the corresponding exchange current densities, extracted from these fits, were highest for the AmLA electrode. This enhancement indicates markedly improved intrinsic charge transfer kinetics at the electrode interface. Consistent with these findings, the symmetric cell CV responses (Figure S15) also demonstrate that AmLA enables stronger redox currents than PVDF and SBR/CMC, corroborating its superior electrocatalytic activity [49].

These results collectively indicate that the amide and lithium acrylate groups within the AmLA architecture stabilize intermediate polysulfide species and effectively lower the activation energy barrier for their conversion to Li_2_S_2_/Li_2_S. This dual functionality suppresses shuttle‐driven active material loss while accelerating interfacial redox reactions, consequently establishing a favorable kinetic environment for highly reversible cycling in Li–S batteries.

### 2.8 Electrochemical Performance

**FIGURE 8:**
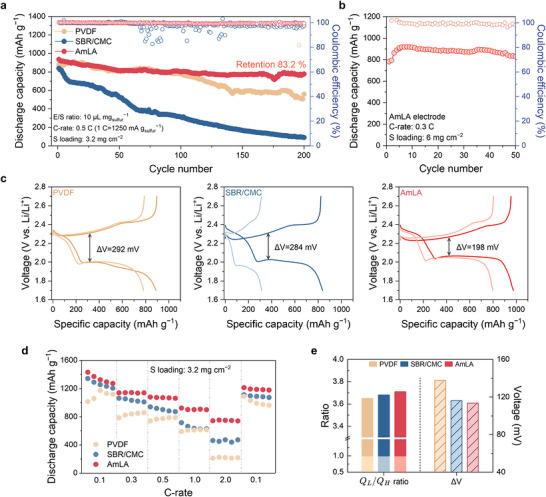
Electrochemical performance of sulfur cathodes evaluated under practical cell conditions with different binder systems. (a) Cycling performance and Coulombic efficiency at 0.5 C with a sulfur loading of 3.2 mg cm^−2^, highlighting the superior retention (83.2%) of the AmLA electrode. (b) Cycling stability test at 0.3 C under a high sulfur loading of 6 mg cm^−2^, demonstrating the robust operation of the AmLA binder in practical regimes. (c) Galvanostatic charge–discharge voltage profiles corresponding to the first (light line) and 50th (solid line) cycles at 0.5 C. The AmLA electrode maintains a stable voltage plateau with minimal polarization increase (Δ*V* = 198 mV) even after prolonged cycling, whereas the PVDF and SBR/CMC electrodes exhibit noticeable capacity decay and voltage hysteresis expansion. (d) Rate capability assessment from 0.1 to 2 C at 3.2 mg cm^−2^, demonstrating the higher discharge capacity of the AmLA cathode. (e) Quantitative comparison of Q_L_/Q_H_ ratios and polarization values (ΔV). The higher Q_L_/Q_H_ ratio for AmLA indicates enhanced sulfur utilization and efficient conversion kinetics during the discharge process.

The electrochemical viability of sulfur cathodes employing the AmLA binder was systematically evaluated against conventional benchmarks under various operating conditions (Figure 8). In the long‐term cycling evaluation at 0.5 C with a sulfur loading of 3.2 mg cm^−2^ (Figure 8a), the AmLA electrode delivered a high initial capacity of 935 mAh g^−1^ and retained 777 mAh g^−1^ after 200 cycles, whereas the SBR/CMC and PVDF electrodes showed rapid capacity fading with significantly lower retention rates. Post‐cycling cross‐sectional SEM analysis further corroborates the structural retention of the AmLA electrode, which maintained a continuous and well‐adhered morphology without the interfacial delamination observed in the PVDF and SBR/CMC electrodes (Figure S18). To assess the feasibility under practical high‐energy conditions, the sulfur loading was increased to 6 mg cm^−2^. Even under this demanding regime, the AmLA electrode maintained a robust capacity of 831 mAh g^−1^ after 50 cycles at 0.3 C (Figure 8b), demonstrating its exceptional stability for practical applications [50].

The galvanostatic charge–discharge voltage profiles at 0.5 C (Figure 8c) further revealed that the AmLA electrode exhibited significantly suppressed polarization, with a voltage gap of 198 mV, compared to 284 mV for SBR/CMC and 292 mV for PVDF. This reduced overpotential indicates more efficient charge–discharge reversibility and faster reaction kinetics in the AmLA‐based cathode. Rate capability tests conducted from 0.1 to 2 C at 3.2 mg cm^−2^ (Figure 8d) also highlighted the superiority of the AmLA binder. The electrode retained a capacity of 748 mAh g^−1^ even at a high rate of 2 C, whereas the SBR/CMC and PVDF electrodes delivered only 472 and 220 mAh g^−1^, respectively.

The analysis of the capacity contribution ratio (Q_L_/Q_H_, representing the ratio of lower‐plateau to higher‐plateau capacity, Figure S19) and the corresponding polarization values (Figure 8e) indicates that the AmLA electrode promotes more efficient sulfur utilization during the discharge process. By minimizing the loss of soluble species into the electrolyte, AmLA ensures a higher conversion efficiency from polysulfides to solid Li_2_S. This trend is consistent with the mechanical robustness, strong polysulfide adsorption capability, and improved ion transport revealed in the preceding analyses. Collectively, these findings demonstrate that the AmLA binder provides a practical pathway to realizing high‐loading lithium–sulfur cathodes with long‐term durability and high energy efficiency. This superior performance originates from the rational molecular design of AmLA, in which the amide groups strengthen adhesion and mechanical integrity, while the lithium acrylate moieties facilitate Li^+^ transport and strong polysulfide binding. This multifunctional synergy ensures electrode stability, efficient sulfur utilization, and reliable cycling performance even under practically relevant high‐loading conditions.

## 3 Conclusion

In this study, we have successfully demonstrated the efficacy of AmLA, a strategically designed water‐processable binder, as a comprehensive solution to the intrinsic challenges of Li–S batteries. The incorporation of AmLA into sulfur cathodes validates the hypothesis that a bifunctional binder architecture can simultaneously address mechanical instability and the polysulfide shuttle effect. The polar amide functionalities reinforce interfacial adhesion and structural cohesion, establishing a robust and porous electrode framework capable of accommodating drastic volume changes during repeated cycling. Concurrently, the ionic lithium acrylate moieties significantly enhance Li^+^ transport kinetics and serve as effective chemical anchors for soluble polysulfides, suppressing their outward diffusion into the electrolyte.

Beyond physical stabilization, our findings reveal that AmLA fundamentally alters the reaction kinetics of the sulfur cathode. By lowering the nucleation energy barrier, the binder promotes the uniform 3D deposition of Li_2_S and accelerates the sluggish liquid–solid phase transformation. This synergistic interplay of mechanical robustness, chemical confinement, and electrocatalytic activity translates into superior electrochemical performance. Under a high sulfur content of 75 wt% with an areal loading of 3.2 mg cm^−2^, the AmLA electrode delivered a discharge capacity of 777 mAh g^−1^ after 200 cycles at 0.5 C, corresponding to an exceptional capacity retention of 83.2%. Furthermore, even under a practically rigorous areal loading of 6.0 mg cm^−2^, the electrode maintained a high reversible capacity of 831 mAh g^−1^ after 50 cycles, confirming its scalability for high‐energy applications.

In summary, the optimized balance of amide and lithium acrylate groups within AmLA underpins a multifunctional mechanism that ensures both structural integrity and efficient sulfur utilization. Overall, the optimized AmLA copolymer provides a composition‐controlled binder design that balances acrylamide‐derived mechanical reinforcement with lithium acrylate‐associated polysulfide regulation and redox‐kinetic improvement, offering a practical route toward durable, high‐loading lithium–sulfur batteries.

## 4 Experimental Section

### 4.1 Synthesis and Characterization of AmLA Binder

The AmLA binder was synthesized via the base‐catalyzed hydrolysis of polyacrylamide (M_w_ = 400,000–800,000, Tokyo Chemical Industry Co., Ltd.) using lithium hydroxide (LiOH). In this reaction, LiOH serves as both the hydrolysis catalyst and the source of lithium ions (Figure S20). The degree of hydrolysis was controlled by adjusting the molar ratio of the PAM repeating units to LiOH. For the synthesis of AmLA with a target hydrolysis degree of 33 mol%, 5 g of PAM and 0.57 g of LiOH were dissolved in 100 mL of deionized water. The mixture was stirred at 80°C for 2 h within a 300 mL reactor. The resulting product was purified by precipitation in an anti‐solvent, followed by vacuum filtration to eliminate residual salts. The purified precipitate was subsequently dried in a vacuum oven at 60°C for 12 h. The chemical structure of the synthesized AmLA was validated using Fourier‐transform infrared spectroscopy (FTIR, IRTracer‐100, Shimadzu) and nuclear magnetic resonance spectroscopy (NMR, AVANCE III 700, Bruker).

### 4.2 Preparation of Sulfur/Carbon Composite and Cathodes

The sulfur/multi‐walled carbon nanotube (MWCNT, S/C) composite was prepared via a melt‐infiltration method [51]. Sulfur powder (≥99.5%, Sigma‐Aldrich) and MWCNTs (CM‐280, Hanwha Chemical Co., Ltd.) were mixed at a weight ratio of 94:6, and homogenized using a ball mill at 200 rpm for 1 h. The mixture was sealed in an argon‐filled Teflon vessel and heated at 155°C for 12 h in a box furnace to facilitate the infiltration of molten sulfur into the MWCNT bundles. To refine the particle size, the resulting composite underwent wet milling in deionized water using a planetary ball mill (XQM‐2A, Changsha Tian Chuang Powder Technology Co., Ltd.) at 500 rpm for 36 h. The pulverized S/C composite was collected via vacuum filtration and dried at 60°C for 6 h. The final sulfur content of the composite was determined using thermogravimetric analysis (TGA, STA8122, Rigaku Co., Ltd.) performed under N_2_ atmosphere at a heating rate of 10°C min^−1^ (Figure S21). For the preparation of sulfur electrodes, the S/C composite, carbon black (CB, Super‐P, Imerys), and the binder were mixed in a weight ratio of 80:10:10. The resulting electrode possessed a high sulfur content of approximately 75 wt%. The slurry was cast onto an aluminum current collector using a doctor blade and dried under vacuum at 60°C for 12 h to remove the solvent. The electrodes were punched into disks with a diameter of 12 mm. The average sulfur loading was measured to be 3.2 mg cm^−2^, corresponding to an areal capacity of 4 mAh cm^−2^.

### 4.3 Electrochemical Measurements

The electrochemical performance of Li–S cells was evaluated using CR2032 coin‐type cells assembled in a dry room maintained at a dew point between −50°C and −60°C. Lithium metal foil (200 µm thick, Honjo Metal Co., Ltd.) served as the anode and was punched into disks with a diameter of 14 mm. A functionalized separator was prepared by blade‐casting a slurry containing Super‐P carbon onto a pristine polyethylene separator (W‐Scope Korea Co., Ltd.). The electrolyte consisted of 0.5 m lithium bis(trifluoromethanesulfonyl)imide (LiTFSI) and 0.5 M lithium nitrate (LiNO_3_) dissolved in a binary solvent mixture of 1,2‐dimethoxyethane (DME) and 1,3‐dioxolane (DOL) (1:1 v/v). The electrolyte‐to‐sulfur ratio was maintained at 10 mL g_sulfur_^−1^.

Galvanostatic charge–discharge tests were conducted within a voltage window of 1.7–2.7 V vs. Li/Li^+^ using a battery cycler (WBS300L, WonATech Co., Ltd.) at 25°C. The standard cycling protocol included a formation step of 5 cycles at 0.1 C, followed by long‐term cycling at 0.5 C (where 1 C was defined as 1250 mA g^−1^). GITT measurements were performed at a current rate of 0.1 C to analyze the thermodynamic equilibrium voltages and kinetic overpotentials. The protocol involved applying a discharge/charge pulse for 10 min, followed by a 2 h relaxation period to allow the cell voltage to stabilize. Cyclic voltammetry (CV) was conducted using a potentiostat (VMP3, BioLogic) over the potential range of 1.7–2.7 V, with scan rates varying from 0.01 to 0.05 mV s^−1^. EIS spectra were acquired using the same instrument over a frequency range of 1 MHz to 0.01 Hz, with an AC perturbation amplitude of 10 mV.

### 4.4 Polysulfide Adsorption Test and In situ UV–vis Spectroscopy

To evaluate the polysulfide adsorption capability, a stoichiometric Li_2_S_6_ solution (2.5 mM) was prepared by chemically reacting Li_2_S and sulfur powder in a molar ratio of 1:5 in a DME/DOL (1:1 v/v) solvent mixture. The precursor mixture was stirred at 50°C under an argon atmosphere inside a glove box to ensure complete reaction and dissolution. Subsequently, 0.3 g of the respective binder was immersed in 10 mL of the prepared Li_2_S_6_ solution and maintained under static conditions for 24 h to reach adsorption equilibrium. The supernatant was then separated via filtration, and its absorbance spectrum was recorded using a UV–vis spectrophotometer (UV‐3600, Shimadzu) to quantify the residual polysulfide concentration. In situ UV–vis spectroscopy was employed to visualize the real‐time variation in LiPS absorption during electrochemical reactions in Li–S cells. The experimental setup is schematically illustrated in Figure 4b. A sulfur cathode (8 × 20 mm) was welded to a nickel tab, while a lithium metal anode attached to a copper foil served as the counter electrode. These components were assembled in a customized quartz cuvette cell with dimensions of 12.5 × 12.5 × 45 mm. The cell was filled with 2 mL of electrolyte containing 0.5 M LiTFSI in a DME/DOL (1:1 v/v) mixture within an argon‐filled glove box. After hermetic sealing, the cell was mounted onto the UV–vis spectroscopy setup. The galvanostatic discharge process was controlled using a potentiostat (VSP‐300, BioLogic). Concurrently, UV–vis spectra were acquired at 2 min intervals across a wavelength range of 320–550 nm to monitor the evolution of polysulfide species and assess the diffusion regulation capability of the binder.

### 4.5 Li_2_S Nucleation Measurement

The kinetics of Li_2_S nucleation were investigated using carbon paper (CP) electrodes modified with different binders and a lithium polysulfide catholyte. The 0.1 m Li_2_S_8_ catholyte (calculated based on sulfur atoms) was prepared by mixing stoichiometric amounts of Li_2_S and sulfur powder (molar ratio of 1:7). This mixture was dissolved in an electrolyte containing 1 m LiTFSI and 0.2 m LiNO_3_ in a binary solvent of DME/DOL (1:1 v/v) by stirring at 70°C and 400 rpm for 24 h to ensure complete dissolution.

For electrode preparation, the respective binder solutions were drop‐cast onto CP substrates, achieving a loading mass of 1.5 mg cm^−2^. The coated electrodes were vacuum‐dried at 60°C for 12 h and subsequently punched into disks with a diameter of 12 mm. For the nucleation test, CR2032 coin cells were assembled by dispensing 40 µL of the Li_2_S_8_ catholyte onto the working electrode and 10 µL of the blank electrolyte (without polysulfide) onto the counter electrode. The nucleation characteristics were evaluated via chronoamperometry. The protocol involved an initial galvanostatic discharge at 0.112 mA until a cutoff voltage of 2.15 V was reached to consume long‐chain polysulfides, followed by a potentiostatic discharge at 2.09 V until the current decayed to 0.01 mA to monitor the nucleation and growth of Li_2_S.

### 4.6 Microstructural Characterization

The microstructural evolution and cross‐sectional morphology of the fabricated electrodes were characterized using field‐emission scanning electron microscopy (FE‐SEM, S‐4800, Hitachi). To ensure high‐quality imaging of the internal structure without mechanical deformation, the electrode cross‐sections were prepared using an argon ion‐beam cross‐section polisher (IB‐19530CP, JEOL) prior to SEM observation.

### 4.7 Mechanical Characterization of Sulfur Cathodes

To quantitatively evaluate the interfacial adhesion strength of the electrodes, a peel test was conducted using a universal testing machine (UTM, 34sc‐05, Instron). The test specimens were prepared by firmly attaching an adhesive tape (3M 810) onto electrode strips with a width of 10 mm. The measurements were performed in T‐peel mode at a constant peeling speed of 10 mm s^−1^ to record the detachment force between the electrode layer and the current collector. Additionally, the surface hardness of the electrodes was assessed via nanoindentation (Nano Test Vantage Platform, Micro Materials). To ensure statistical reliability, a minimum of 10 independent indentations were performed for each sample. Crucially, to eliminate discrepancies arising from variations in electrode porosity, a controlled calendering process was applied to all samples prior to testing. This standardization of electrode density ensured that the measured mechanical properties reflected the intrinsic binder efficacy rather than differences in particle packing.

## Conflicts of Interest

The authors declare no conflict of interest.

## Supporting information




**Supporting File**: smll73929‐sup‐0001‐SuppMat.docx.

## Data Availability

The data that support the findings of this study are available from the corresponding author upon reasonable request.
