# Supplementary material for: Synergizing Polysulfide Trapping and Fast Ion Kinetics Enabled by Poly(Acrylamide‐co‐Lithium Acrylate) Aqueous Binder for High‐Sulfur‐Loading Lithium–Sulfur Batteries
Source: Small. 2026 May 25;22(39):e73929. doi: 10.1002/smll.73929 (PMC13360691; doi:10.1002/smll.73929)
Supplement: Supplementary file 1 — Supporting File: smll73929‐sup‐0001‐SuppMat.docx. [file SMLL-22-e73929-s001.docx]

Supporting Information

Synergizing Polysulfide Trapping and Fast Ion Kinetics Enabled by Poly(Acrylamide-co-Lithium Acrylate) Aqueous Binder for High-Sulfur-Loading Lithium–Sulfur Batteries

Seongbae Park^1,2^, Moongil Bang^1,2^, Seungmok Kang^1,2^, Seongin Lee^1,2^, and Young-Jun Kim^1,2,3*^

^1^SKKU Advanced Institute of Nano Technology (SAINT), Sungkyunkwan University, Suwon 16419, Republic of Korea

^2^Department of Nano Science and Technology, Sungkyunkwan University, Suwon, 16419, Republic of Korea

^3^SKKU Institute of Energy Science and Technology (SIEST), Sungkyunkwan University, Suwon, 16419, Republic of Korea


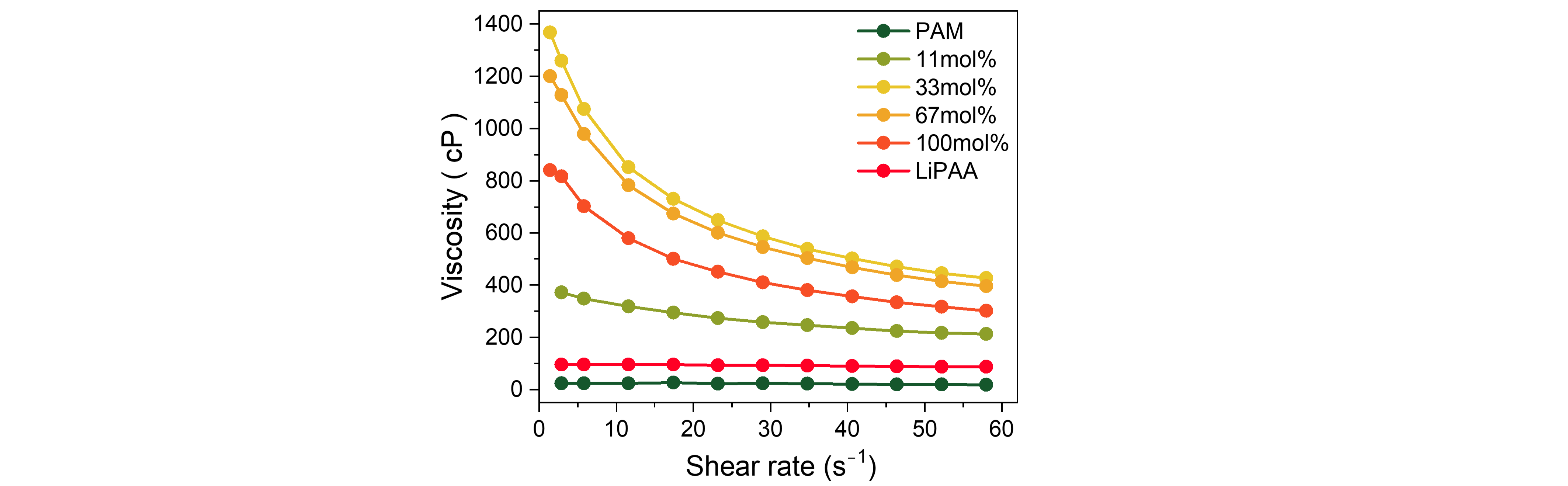


**Figure S1.** Rheological viscosity–shear rate profiles of 1 wt% aqueous binder solutions. All samples exhibit characteristic shear-thinning behavior, which facilitates slurry processing. Notably, the AmLA binder with an intermediate conversion ratio (33 mol%) displays the highest viscosity compared to pristine PAM and fully converted LiPAA reference. This peak in viscosity indicates the formation of a strong intermolecular network arising from the synergistic combination of hydrogen bonding and ionic interactions.


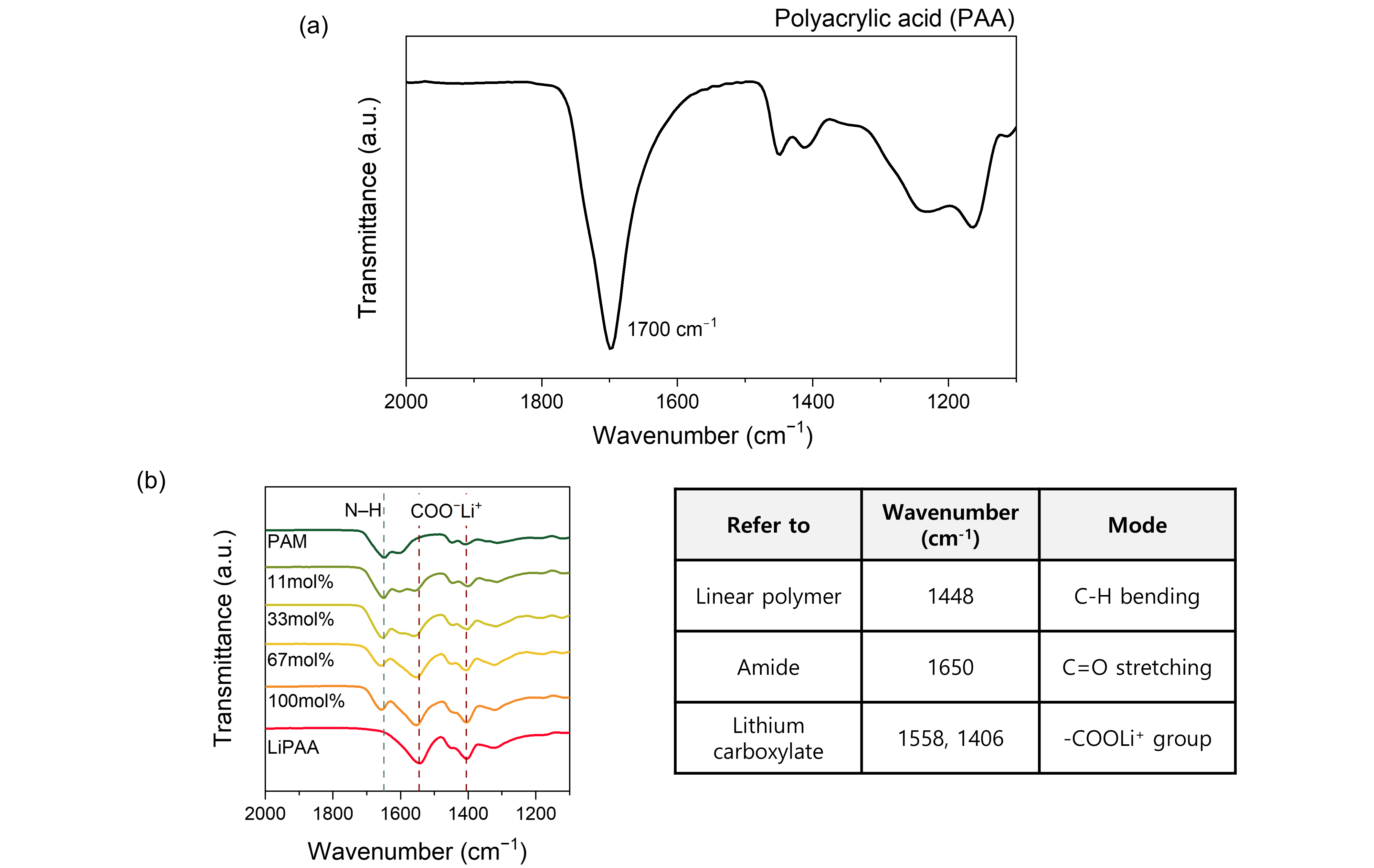


**Figure S2.** Fourier transform infrared (FTIR) spectra illustrating the chemical structural evolution of the binders. (a) Spectrum of the PAA reference. (b) Comparative spectra of pristine PAM, AmLA binders synthesized with varying degrees of hydrolysis (11–100 mol%), and the fully converted LiPAA. The characteristic absorption bands are assigned to the amide carbonyl stretching at 1650 cm^−1^, lithium carboxylate symmetric and asymmetric stretching at 1558 and 1406 cm^−1^, and C–H bending vibration of the linear polymer backbone at 1448 cm^−1^.


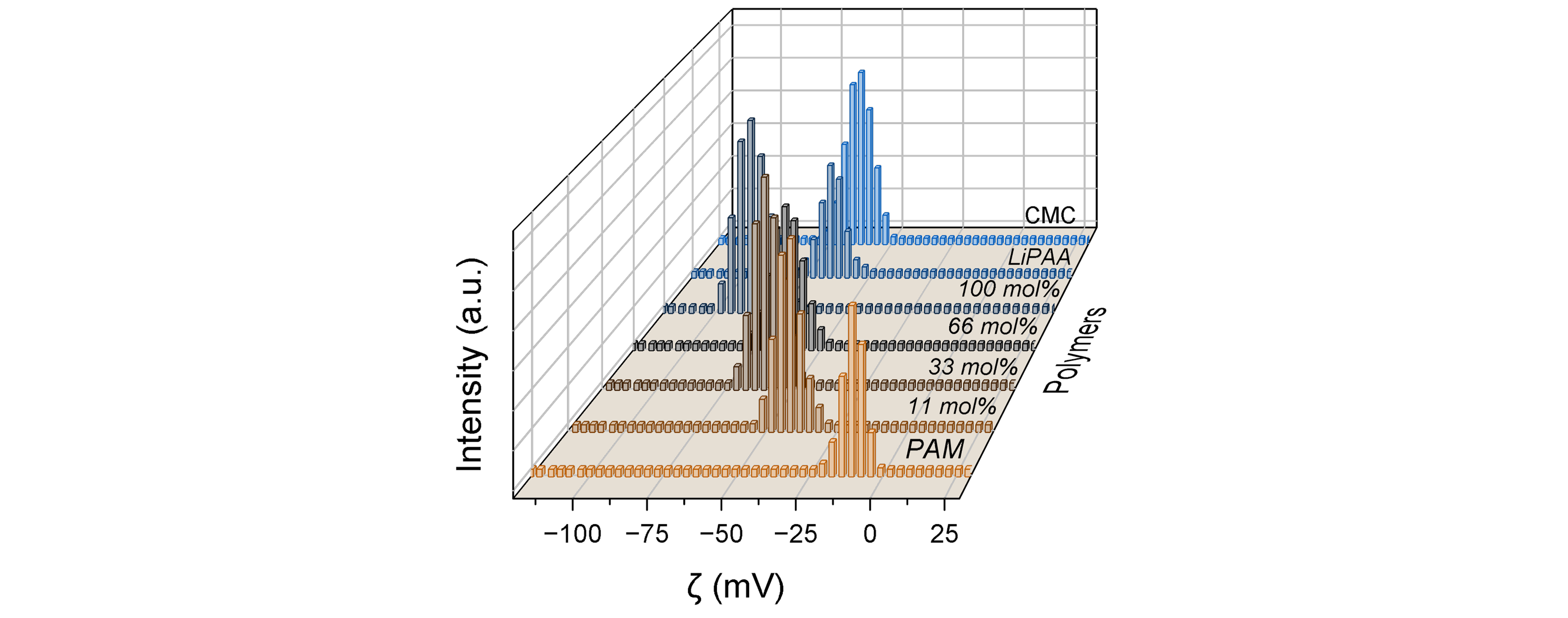


**Figure S3.** Zeta potential distribution profiles of pristine PAM, AmLA variants with varying degrees of hydrolysis (11–100 mol%), fully converted LiPAA, and the carboxymethyl cellulose (CMC) reference measured at neutral pH. The progressive shift toward more negative potentials with increasing lithium acrylate content confirms the enhancement in colloidal stability driven by electrostatic repulsion.


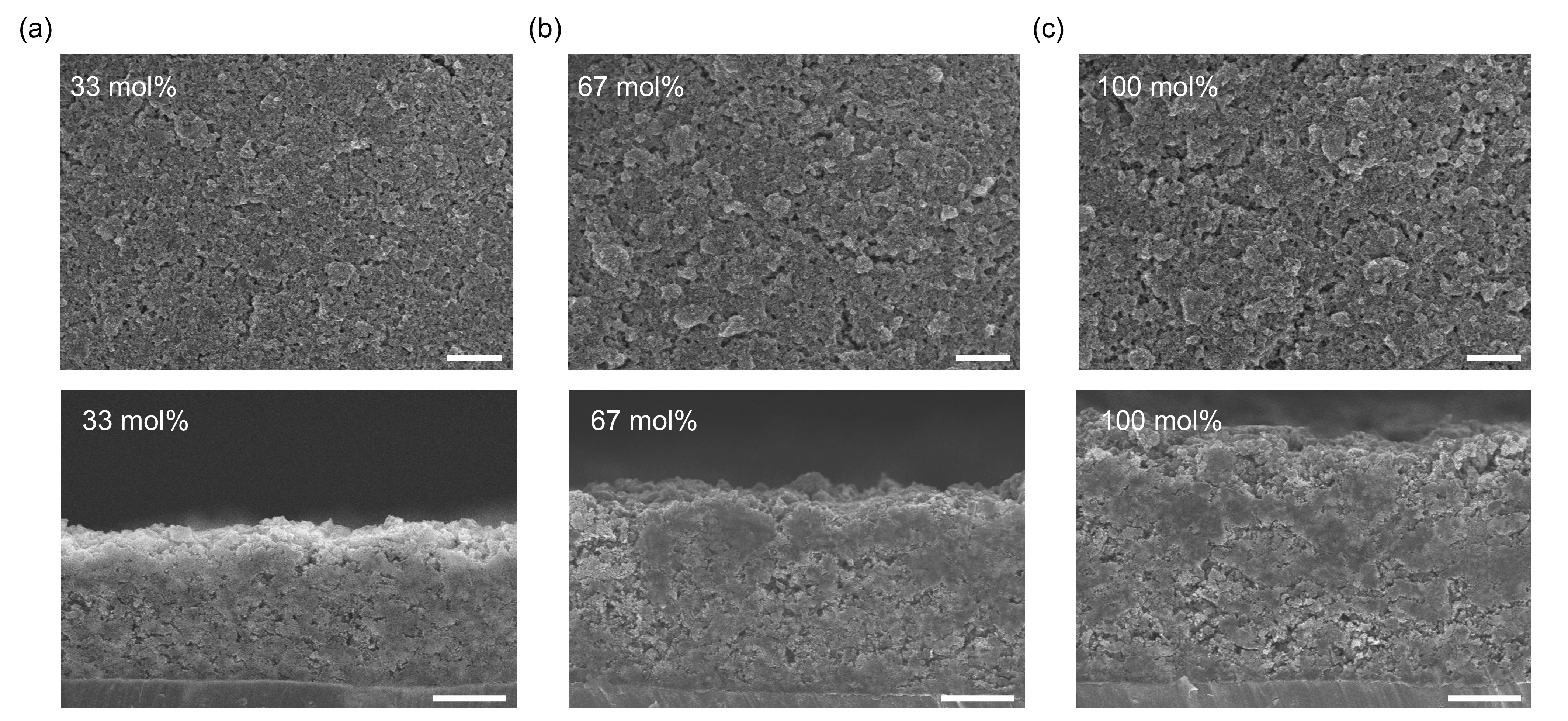


**Figure S4.** Scanning electron microscopy (SEM) images illustrating the morphological evolution of the surface (top row) and cross-section (bottom row) of sulfur cathodes with varying binder compositions. The panels display electrodes prepared with (a) 33 mol%, (b) 67 mol%, and (c) 100 mol% AmLA binders. The electrode employing 33 mol% AmLA exhibits a uniform and cohesive morphology in both views, indicative of optimal mechanical integrity and uniform component dispersion. Conversely, electrodes with higher conversion ratios display increased defects and a less compact structure, a trend attributed to the reduced density of hydrogen-bonding amide groups. The images are presented at magnifications of ×300 and ×1,000, and all scale bars correspond to 50 μm.


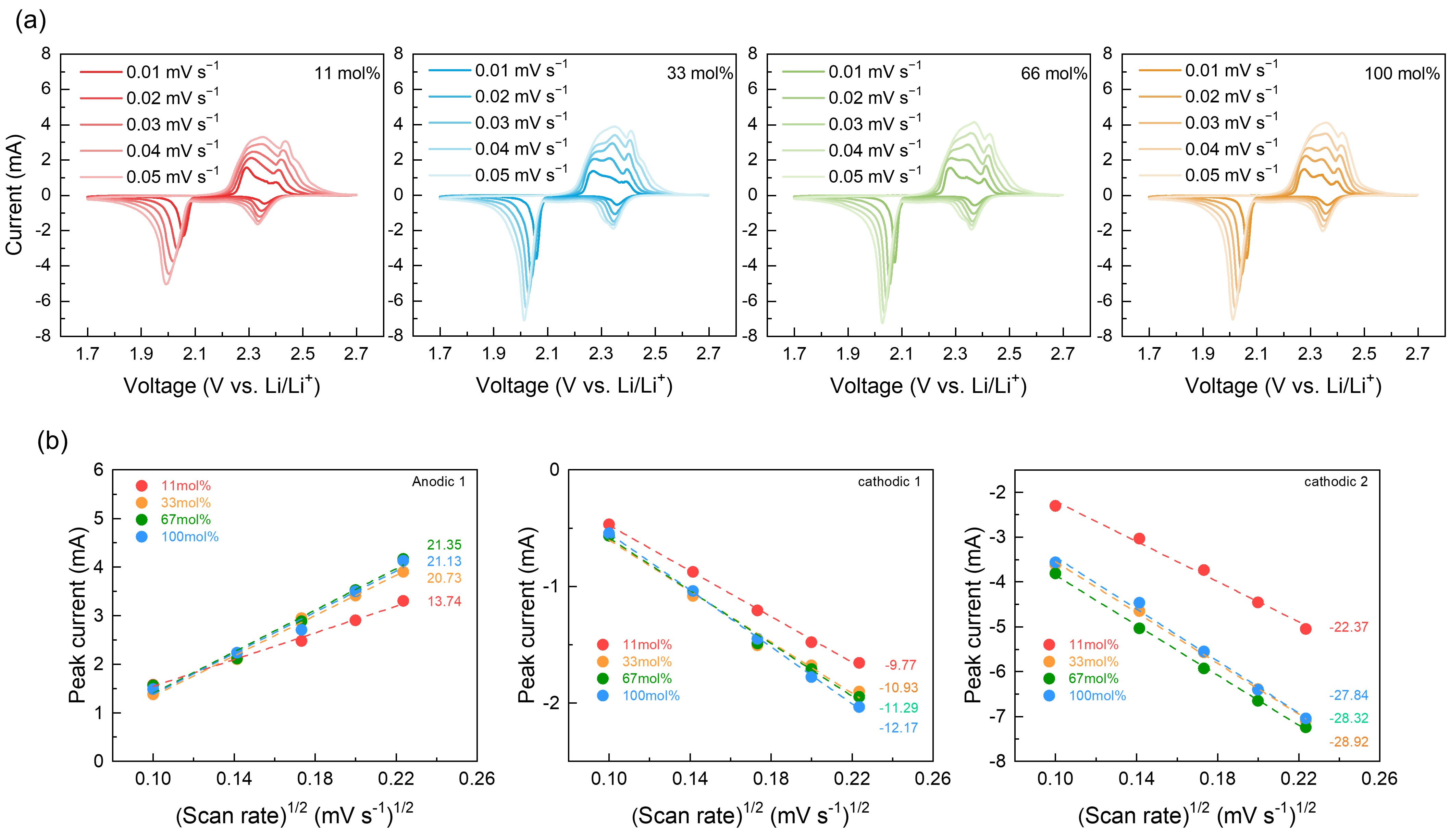


**Figure S5.** Electrochemical kinetic analysis of sulfur cathodes employing AmLA binders with varying hydrolysis ratios. (a) Representative cyclic voltammetry (CV) profiles recorded at scan rates ranging from 0.01 to 0.05 mV s^−1^. (b) Linear fitting of the peak current versus the square root of the scan rate ν^1/2^ corresponding to the major redox peaks. These plots are derived using the Randles–Sevcik equation to estimate the apparent lithium-ion diffusion coefficients. The results reveal that the AmLA binders exhibit comparable slopes across the different conversion ratios. This finding demonstrates that the 33 mol% formulation effectively maintains rapid lithium-ion transport kinetics equivalent to those of higher conversion ratios, despite its lower lithium carboxylate content.


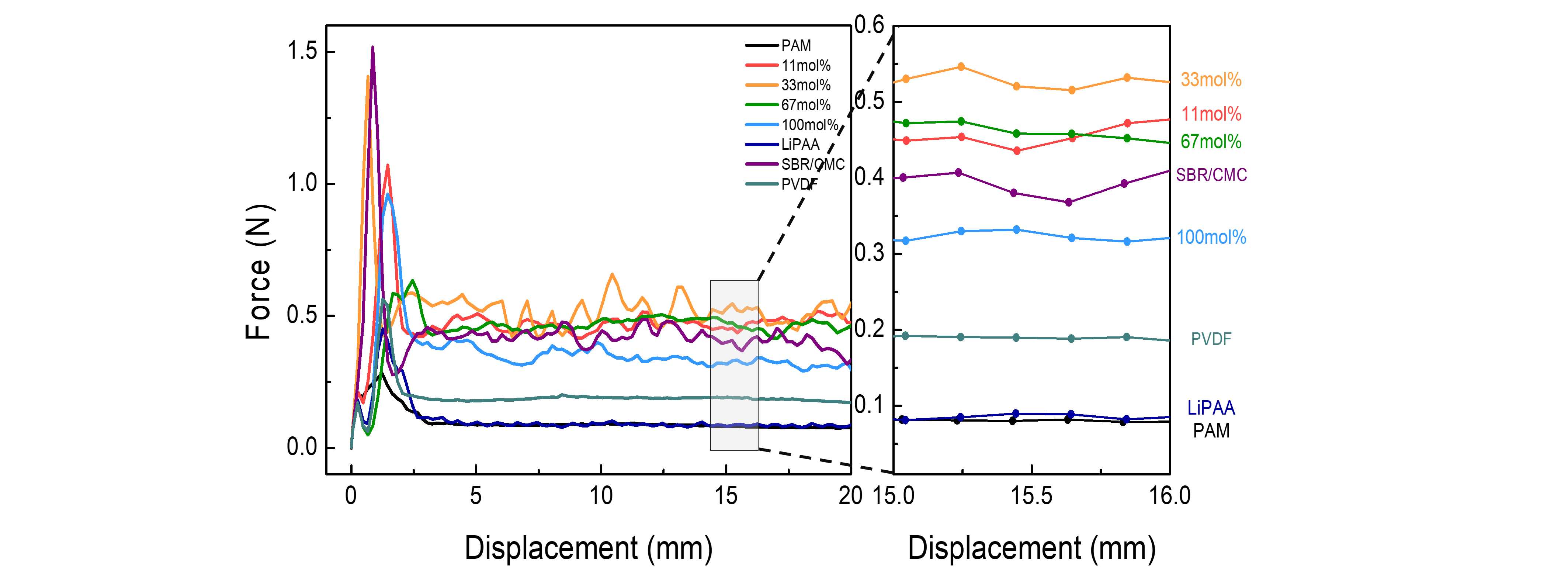


**Figure S6.** Comparative peel strength evaluation of sulfur electrodes prepared with different binder systems, including AmLA with varying conversion ratios and commercial benchmarks (styrene-butadiene rubber/carboxymethyl cellulose (SBR/CMC) and polyvinylidene fluoride (PVDF)). The right panel provides a magnified view corresponding to the gray shaded region to clearly distinguish the steady-state peeling behavior. These results demonstrate that the partially hydrolyzed AmLA binders exhibit superior adhesive strength compared to the fully converted LiPAA and conventional counterparts, confirming the essential contribution of the residual amide groups to the mechanical robustness of the electrode.


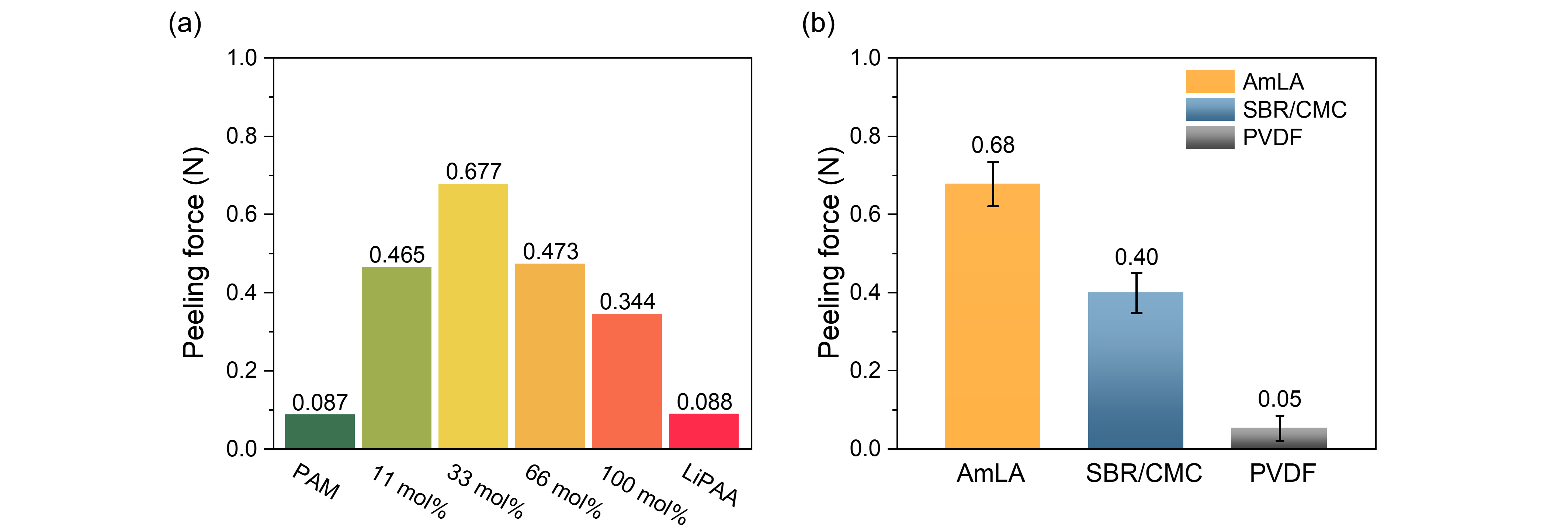


**Figure S7.** Quantitative comparison of electrode adhesion strength derived from T-peel tests. (a) Average peeling forces of sulfur cathodes prepared with AmLA binders varying in hydrolysis degree. The data reveal a distinct "volcano-shaped" trend where the adhesive strength peaks at an intermediate conversion of 33 mol% (0.677 N), significantly surpassing both pristine PAM (0.087 N) and fully converted LiPAA (0.088 N). This confirms that a balanced ratio of hydrogen-bonding amide groups and ionic carboxylate groups is essential for mechanical integrity. (b) Benchmarking the adhesive strength of the optimized AmLA (33 mol%) against commercial binders. The AmLA electrode exhibits a peeling force of approximately 0.68 N, which is 1.7 times higher than that of SBR/CMC (0.40 N) and over 13 times higher than that of PVDF (0.05 N), demonstrating its superior binding capability.


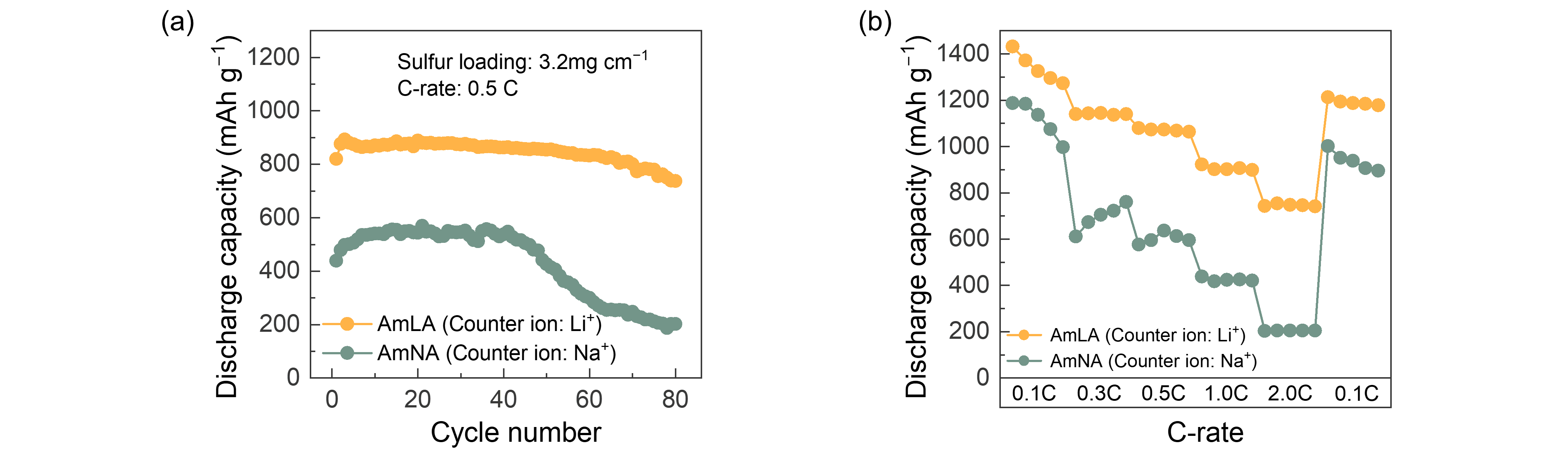


**Figure S8.** Influence of the counter-cation identity on the electrochemical kinetics of sulfur cathodes. (a) Long-term cycling performance evaluated at 0.5 C and (b) rate capability assessment at various C-rates, comparing the standard AmLA binder (synthesized via LiOH, Li^+^ counter-ion) with the sodium-based analogue (synthesized via NaOH, Na^+^ counter-ion). The Li^+^-based AmLA exhibits significantly superior capacity retention and rate response compared to the Na^+^ counterpart. This performance gap highlights the critical role of the lithium carboxylate groups in facilitating facile Li-ion transport pathways, a function that the sodium ions fail to support effectively.


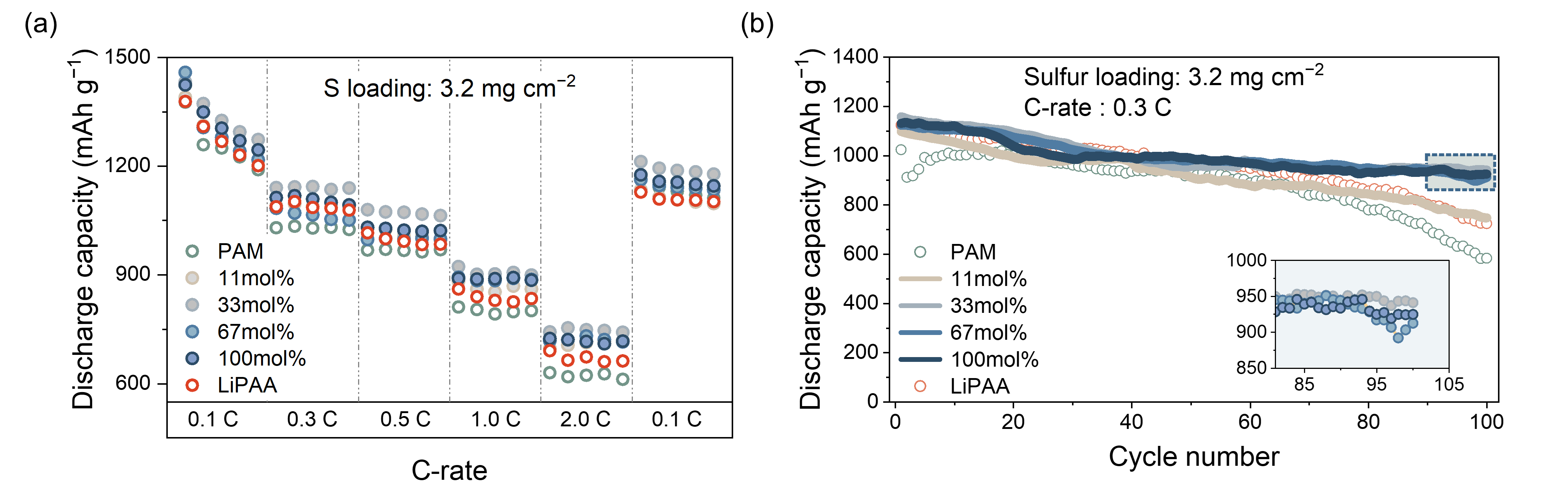
**Figure S9.** Optimization of electrochemical performance as a function of the binder hydrolysis degree. (a) Rate capability evaluated at current densities ranging from 0.1 C to 2.0 C. (b) Cycling performance tested at a constant rate of 0.3 C. All cathodes were prepared with a sulfur loading of 3.2 mg cm^−2^. The comparative results clearly demonstrate that the intermediate conversion ratios (particularly 33 and 67 mol%) deliver superior capacity retention and rate response compared to the pristine PAM and fully converted LiPAA benchmarks. This trend corroborates the necessity of balancing mechanical adhesion (via amide groups) and ionic conductivity (via lithium carboxylate groups) to achieve optimal battery performance. The inset in (b) provides a magnified view of the final cycles to highlight the stability of the optimized formulations.


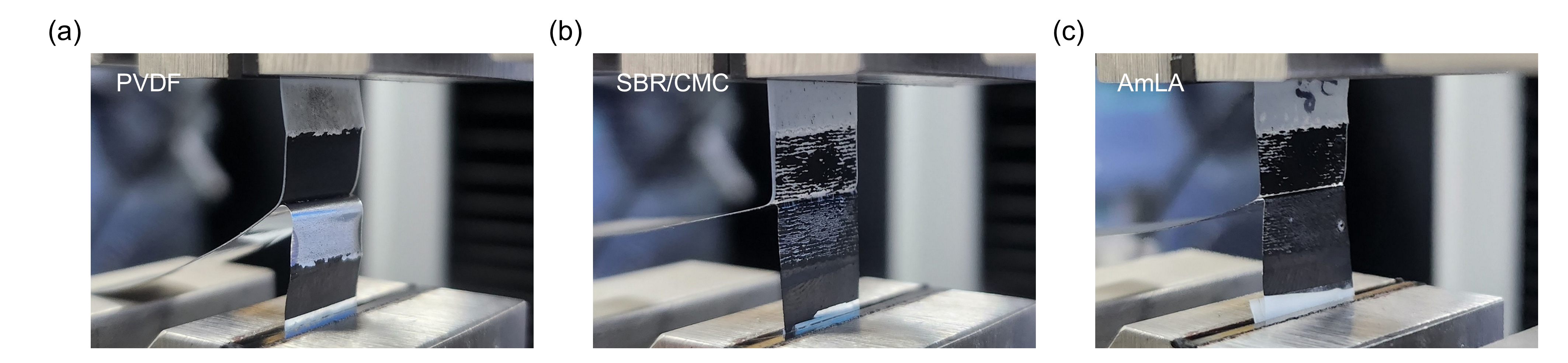


**Figure S10.** Macroscopic visual inspection of electrode integrity following the T-peel adhesion test. The panels display the failure interfaces for (a) PVDF, (b) SBR/CMC, and (c) AmLA electrodes. In each image, the top section corresponds to the adhesive tape peeled from the surface, while the bottom section shows the electrode layer remaining on the current collector. The PVDF and SBR/CMC samples exhibit significant material detachment and exposure of the underlying foil, indicating weak interfacial bonding. In contrast, the AmLA electrode retains a uniform and dense coating on the current collector with minimal delamination. This visual evidence corroborates the quantitative peeling force data, confirming the robust adhesion strength provided by the AmLA binder.


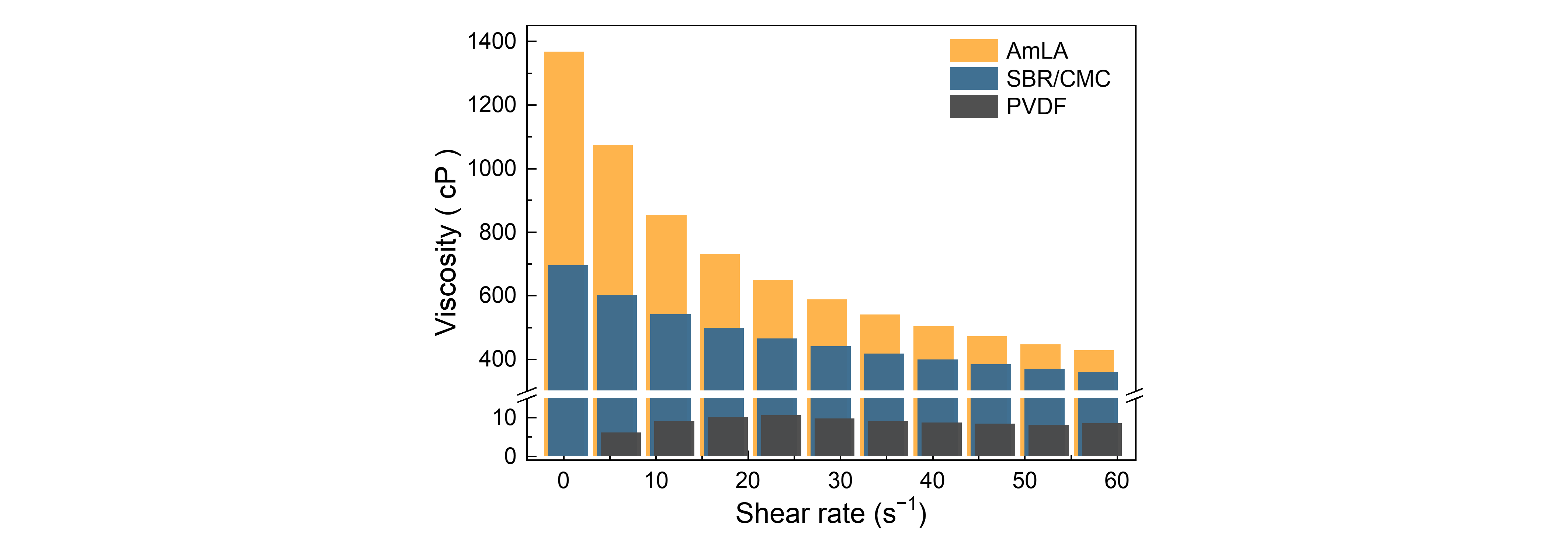


**Figure S11.** Comparison of viscosity values for 1 wt% binder solutions measured at discrete shear rates ranging from 0 to 60 s^−1^. The bar chart illustrates that the AmLA binder possesses the highest initial viscosity (1380 cP), which is favorable for preventing particle sedimentation. With increasing shear rates, the viscosity of AmLA gradually decreases, exhibiting a shear-thinning trend that facilitates smooth coating. In contrast, the PVDF solution shows negligible viscosity values (< 20 cP) across the entire range, as indicated by the axis break. This comparison highlights the superior ability of AmLA to modulate slurry rheology compared to conventional binders.
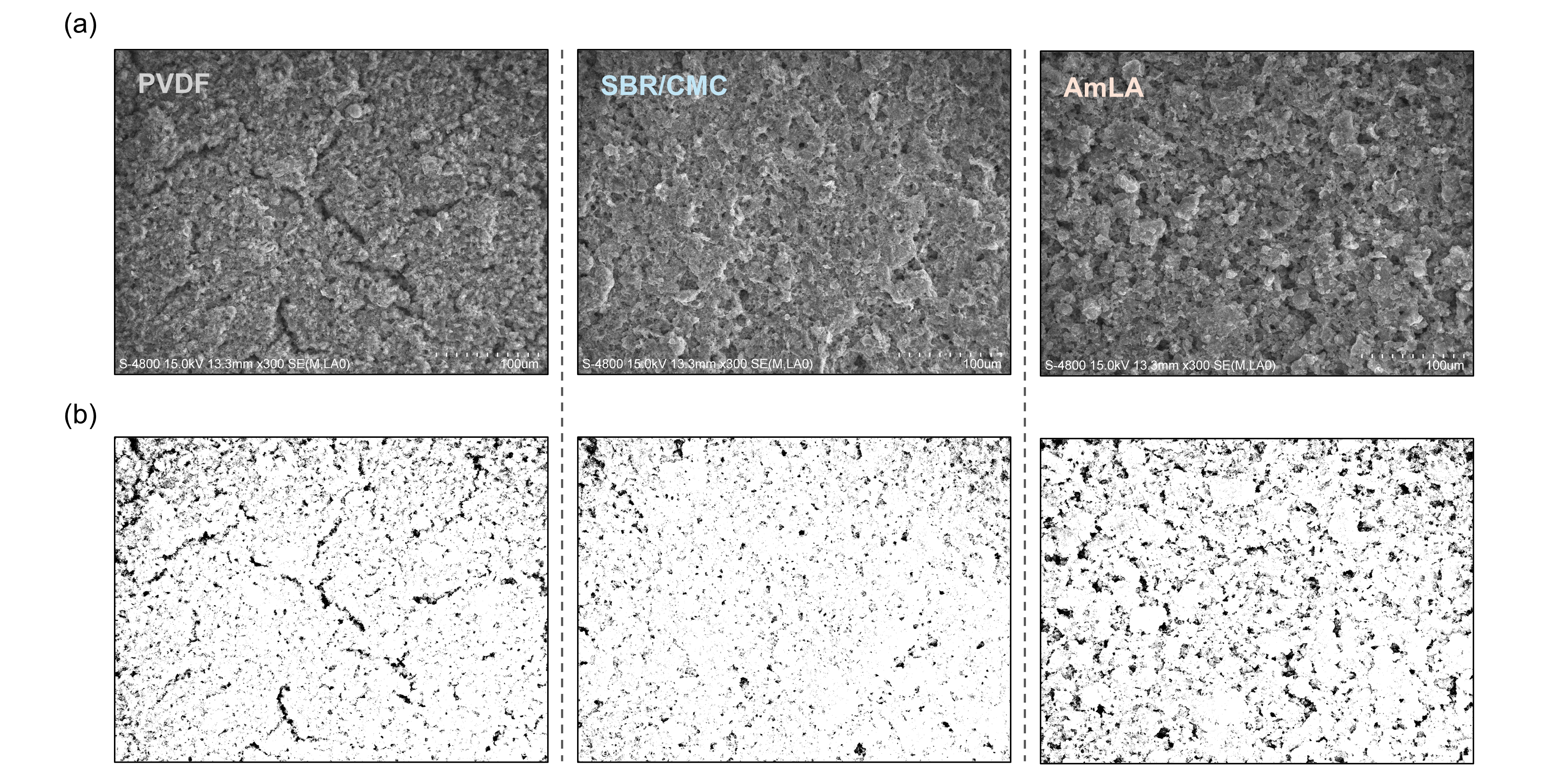


**Figure S12.** Comparative analysis of surface morphology and pore distribution in sulfur cathodes. (a) Top-view SEM images of electrodes prepared with PVDF, SBR/CMC, and AmLA binders. (b) Corresponding binary images processed via color inversion to visualize the surface porosity, where black regions denote pores. The PVDF electrode displays extensive cracking indicative of mechanical failure, while the SBR/CMC electrode exhibits an overly dense structure with limited porosity. In contrast, the AmLA electrode features a homogeneous distribution of pores within a crack-free architecture. This well-developed porous network is essential for facilitating efficient electrolyte infiltration and rapid Li-ion transport.


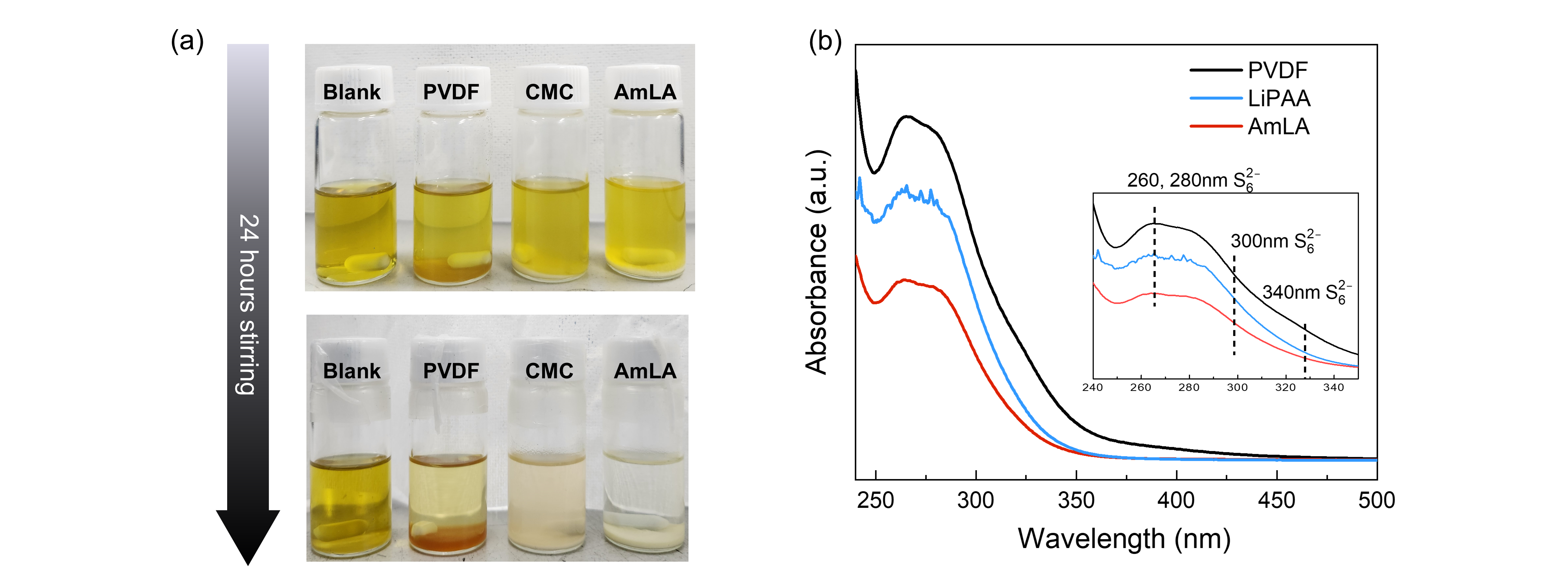


**Figure S13.** Visual and spectroscopic assessment of polysulfide adsorption capability. (a) Digital photographs comparing the color evolution of a 2.5 mM Li_2_S_6_ solution before and after 24 h of exposure to different binder powders. The solution treated with AmLA becomes transparent, indicating effective polysulfide removal, whereas the PVDF and CMC samples retain a distinct yellow coloration. (b) Comparative UV–vis absorption spectra recorded after the adsorption test. The AmLA sample exhibits the lowest absorbance intensity across the characteristic polysulfide region (inset), outperforming both the non-polar PVDF and the fully lithiated LiPAA. This superior adsorption performance is attributed to the dual functionality of the AmLA binder, where the coexistence of amide and lithium carboxylate groups provides abundant active sites for chemically anchoring soluble polysulfide species.


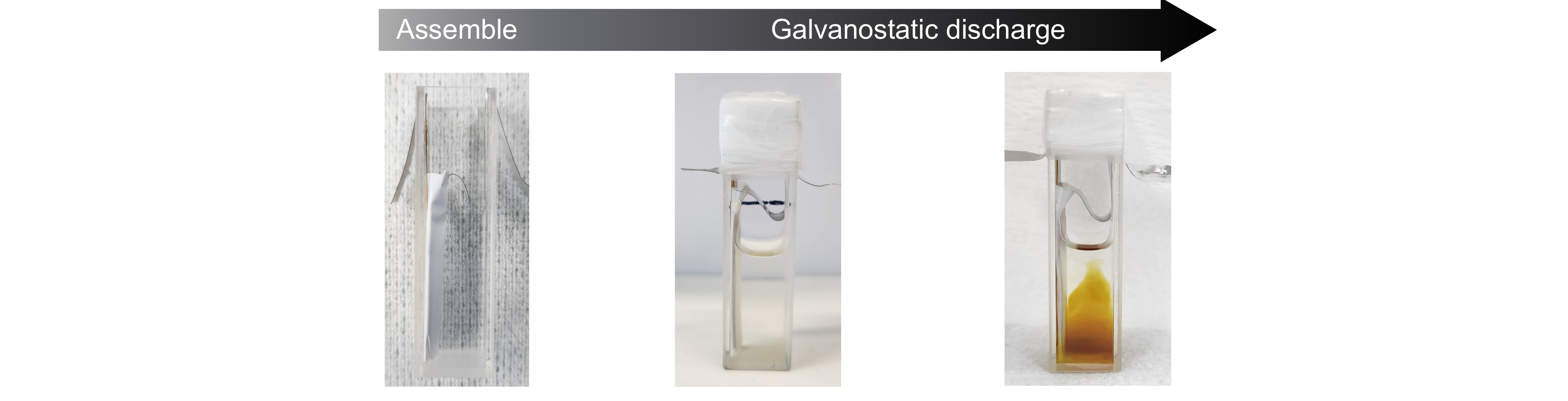


**Figure S14.** Digital photographs illustrating the configuration of the customized quartz cuvette cell utilized for in situ UV–vis spectroscopic analysis. The sequence depicts the transition from cell assembly to the operational state during galvanostatic discharge. The emergence of a distinct yellow hue in the electrolyte provides direct visual confirmation of lithium polysulfide (Li_2_S_x_) dissolution and outward diffusion from the cathode structure. This experimental setup enables the real-time monitoring of the shuttle effect and validates the capacity of the binder to mitigate active material loss.


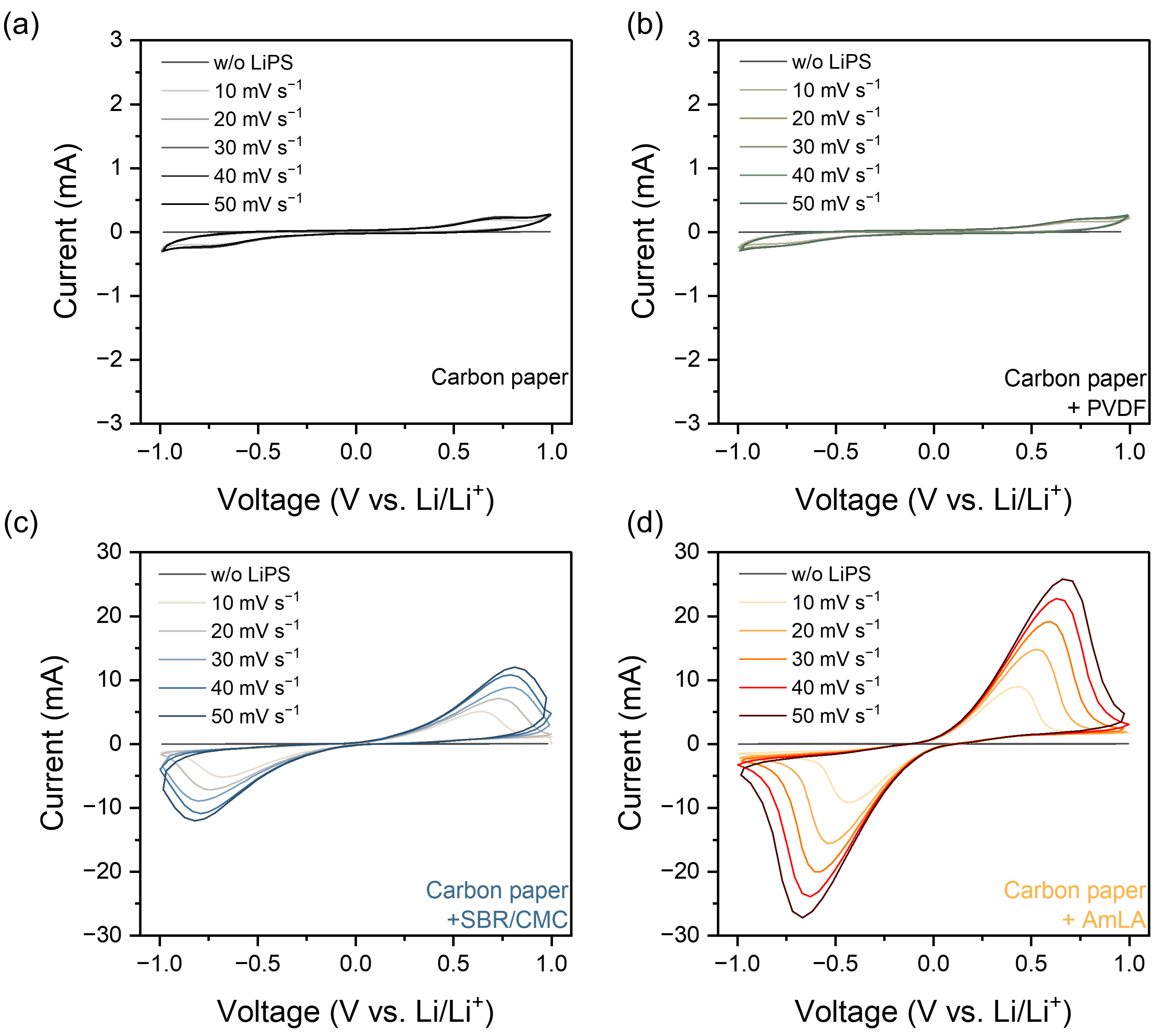


**Figure S15.** Evaluation of polysulfide redox kinetics via symmetric cell [carbon paper (CP) || catholyte || CP] measurements. Cyclic voltammograms (CV) of symmetric cells employing (a) bare CP, (b) CP with PVDF, (c) CP with SBR/CMC, and (d) CP with AmLA as identical working electrodes. The electrolyte consisted of 0.1 M Li_2_S_8_ in DOL/DME (20 μL), and scans were performed at rates ranging from 10 to 50 mV s^−1^ within a voltage window of −1.0 to 1.0 V vs. Li/Li^+^. The bare CP and PVDF-modified electrodes exhibit negligible current responses, indicating sluggish kinetics. In contrast, the AmLA-modified cell delivers the highest redox current density with well-defined peaks, significantly surpassing the SBR/CMC counterpart. This result confirms that the AmLA binder possesses superior electrocatalytic activity, effectively accelerating the reversible conversion of liquid polysulfide species.


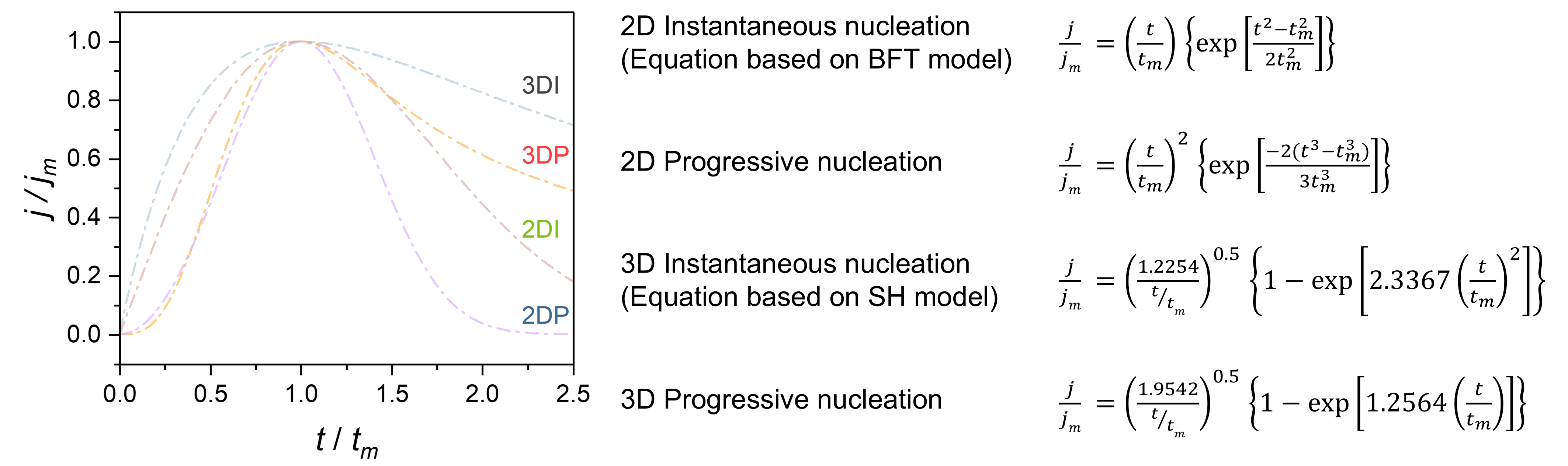


**Figure S16.** Theoretical framework for nucleation kinetics analysis. (Left) Simulated dimensionless current–time (*j*/*j_m_* vs. *t*/*t_m_*) transient profiles representing distinct nucleation and growth regimes: two-dimensional instantaneous (2DI) and progressive (2DP) nucleation, alongside three-dimensional instantaneous (3DI) and progressive (3DP) nucleation. (Right) The corresponding mathematical governing equations derived from the Bewick–Fleischmann–Thirsk (BFT) model for 2D growth and the Scharifker–Hills (SH) model for 3D growth. These theoretical baselines serve as the criteria for classifying the experimental Li_2_S deposition mechanisms observed in the potentiostatic discharge tests.


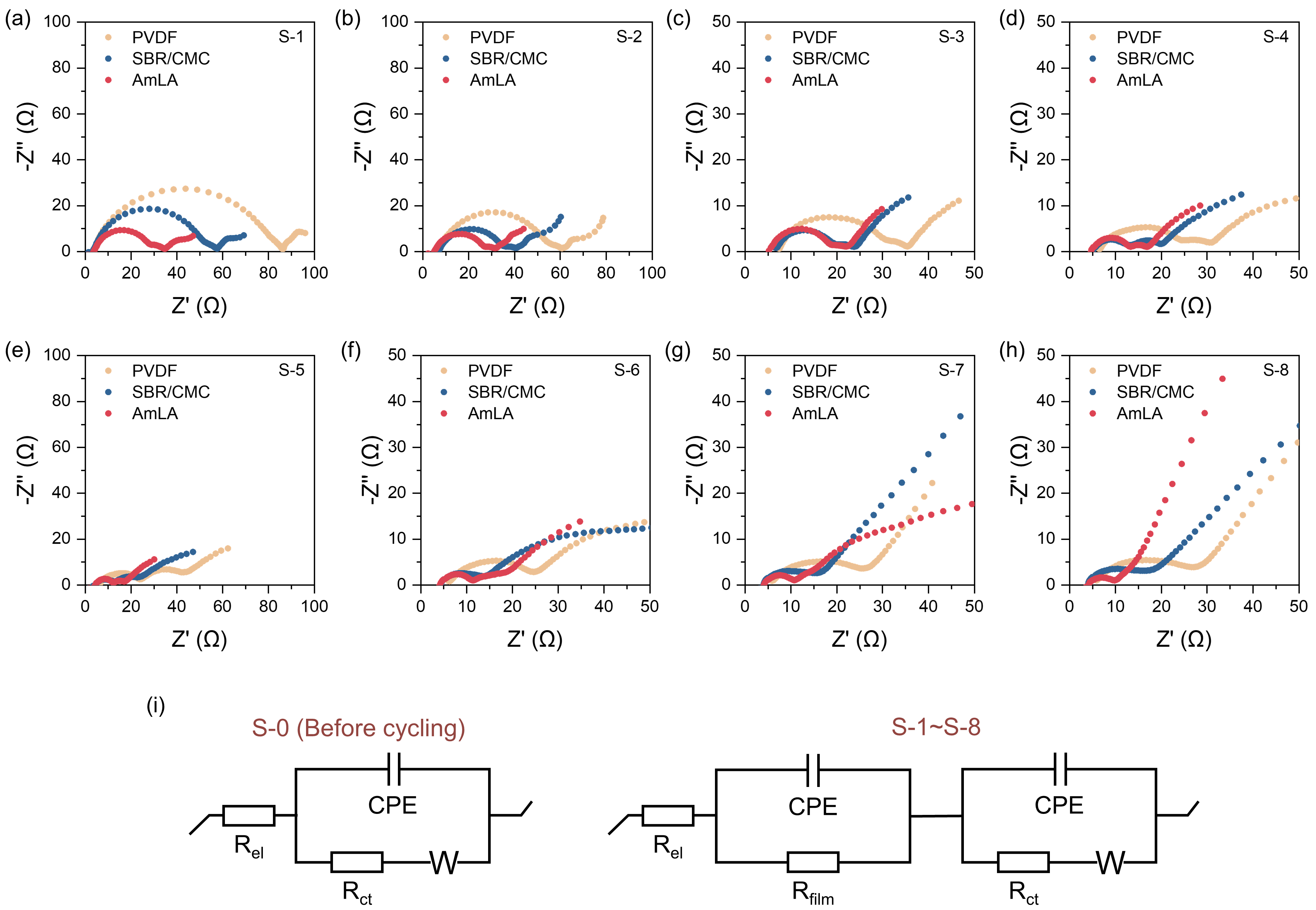
**Figure S17.** Detailed evolution of electrochemical impedance spectra during the discharge process. (a–h) Nyquist plots of Li–S cells employing PVDF, SBR/CMC, and AmLA binders recorded at specific depths of discharge (DoD) corresponding to states S-1 to S-8, as defined in the voltage profile of **Figure 6a**. Throughout the entire discharge depth, particularly in the intermediate polysulfide-conversion region, the AmLA electrode consistently exhibits the smallest semi-circle diameters. This trend indicates significantly reduced interfacial, surface film, and charge-transfer resistances compared to the PVDF and SBR/CMC counterparts. (i) The equivalent circuit model utilized for fitting the impedance spectra, comprising electrolyte resistance (R_el_), surface film resistance (R_film_), charge-transfer resistance (R_ct_), constant phase elements (CPE), and Warburg impedance (W).


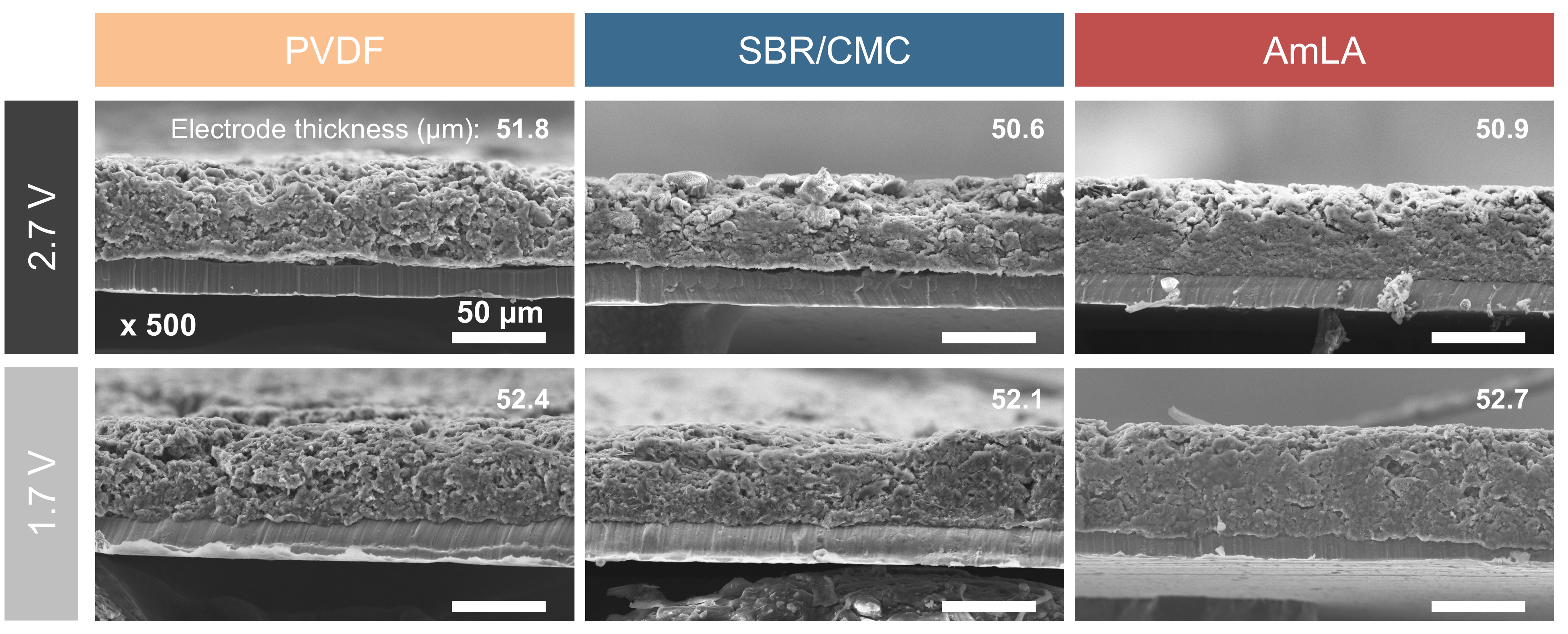
**Figure S18.** Post-cycling cross-sectional SEM images of sulfur cathodes employing PVDF, SBR/CMC, and AmLA binders after 30 cycles at 0.5 C. The cells were disassembled at the charged state of 2.7 V and the discharged state of 1.7 V to compare the state-dependent electrode morphology. The representative electrode thicknesses and cross-sectional morphologies are indicated in each image.


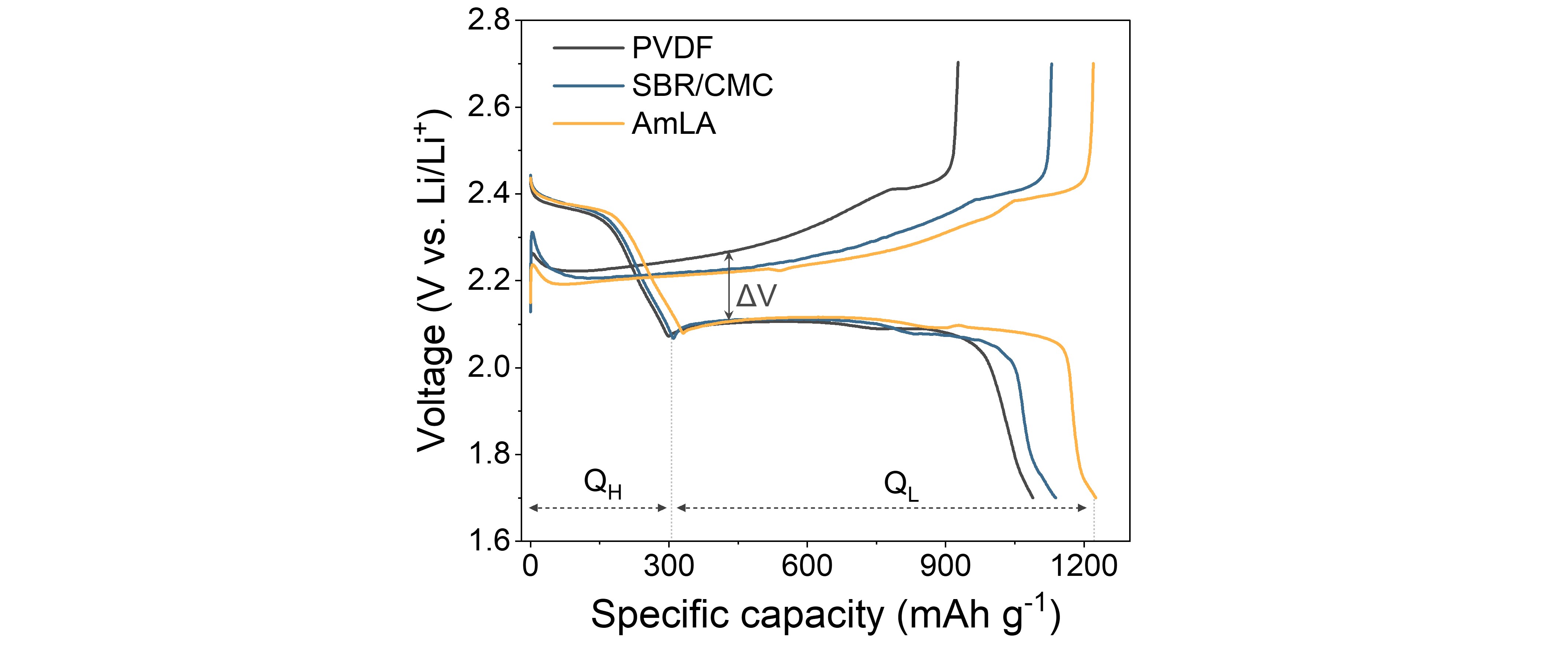


**Figure S19.** Galvanostatic charge–discharge profiles illustrating the capacity contribution of distinct reaction stages. The voltage profiles of sulfur cathodes employing PVDF, SBR/CMC, and AmLA binders were recorded at a current rate of 0.1 C. The specific capacities corresponding to the upper plateau (Q_H_, S_8_ → Li_2_S_4_) and the lower plateau (Q_L_, Li_2_S_4_ → Li_2_S) are demarcated, along with the polarization gap (ΔV). The AmLA electrode exhibits the most extended Q_L_ region combined with the smallest ΔV, demonstrating that the binder effectively promotes the kinetically sluggish liquid–solid phase transformation and maximizes active material utilization.


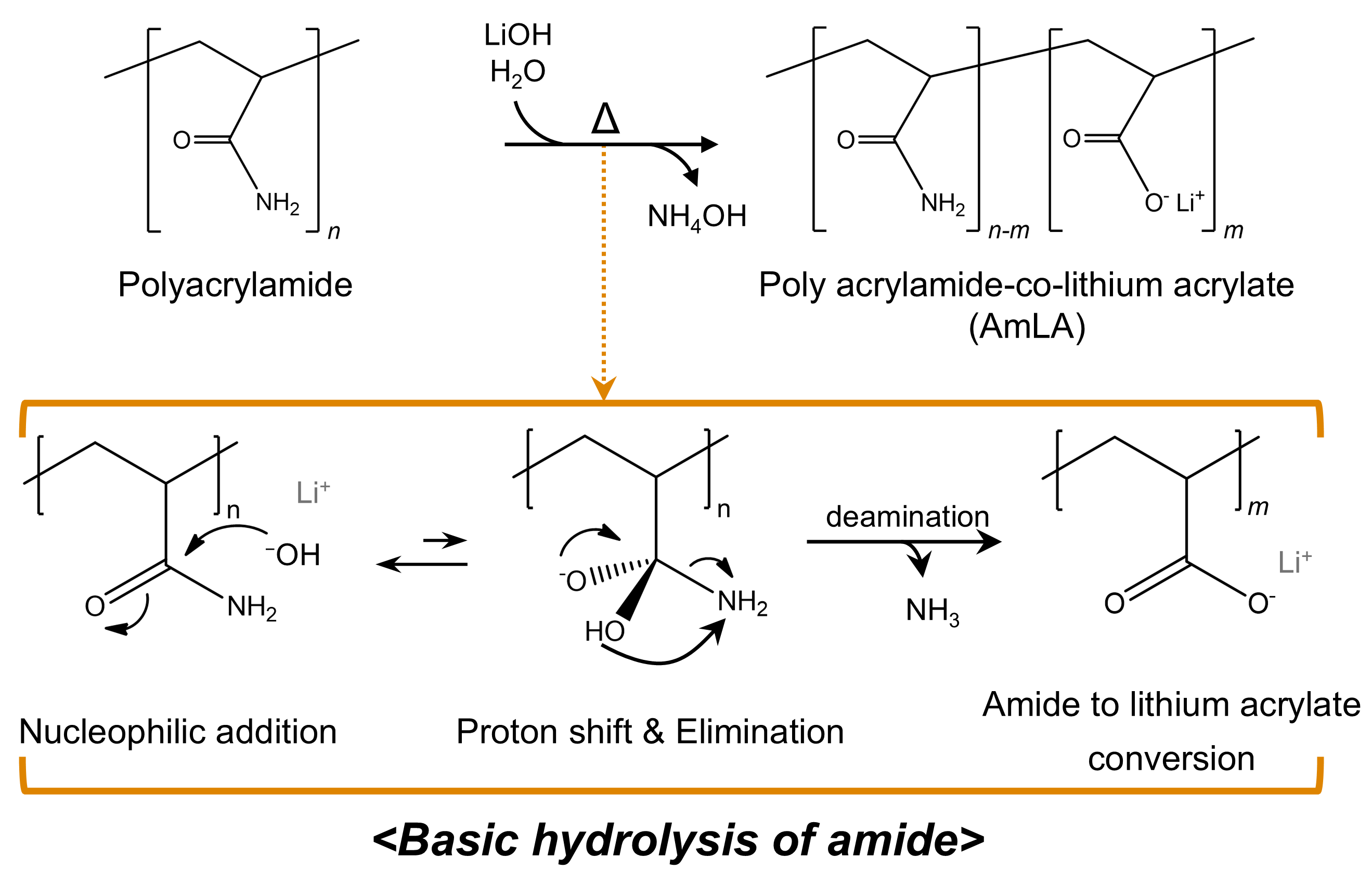


**Figure S20.** Synthetic scheme and mechanistic pathway for the preparation of the AmLA binder. The upper panel depicts the chemical conversion of polyacrylamide (PAM) via basic hydrolysis using lithium hydroxide (LiOH), which introduces lithium acrylate moieties into the polymer backbone. The lower panel details the stepwise reaction mechanism, initiated by the nucleophilic addition of a hydroxide ion (OH^−^) to the amide carbonyl carbon. This is followed by a proton shift and the elimination of ammonia (NH_3_) (deamination), ultimately yielding the stable lithium carboxylate functionality (–COO^−^Li^+^).


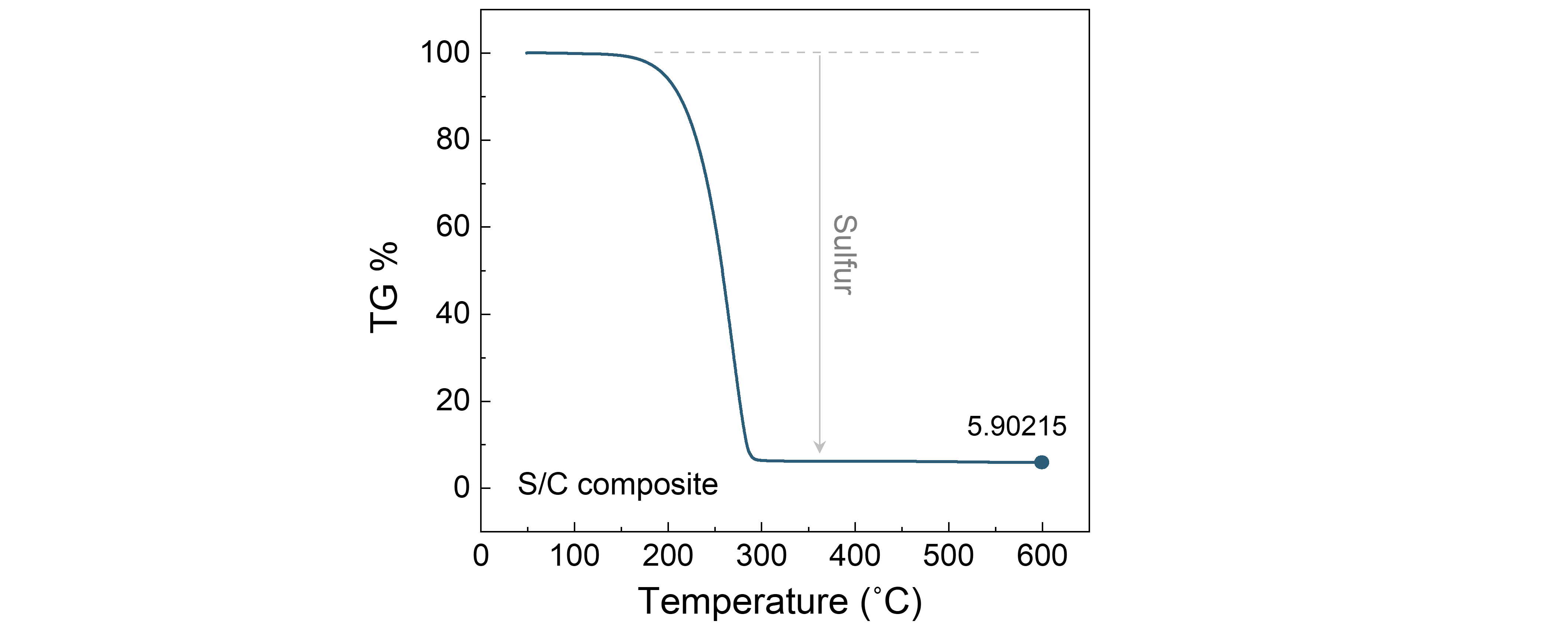


**Figure S21.** Thermogravimetric analysis (TGA) profile of the synthesized sulfur/carbon (S/C) composite measured under a nitrogen atmosphere. The distinct weight loss observed between 200–300 °C corresponds to the sublimation of sulfur. Based on the residual mass of the carbon host (~ 5.9 wt%), the sulfur content within the composite is calculated to be approximately 94 wt%, confirming the precise formulation of the active material via the melt-infiltration process.

**Table S1.** Li^+^ diffusion coefficients for sulfur cathodes with various binders, calculated from Randles–Sevcik plots.^a^

| Peak | Binder | Slope (mA (mV s^−1^)^−0.5^) | D_Li+_ (10^−7^ cm^2^ s^−1^) |
| --- | --- | --- | --- |
| A1 | PVDF | 16 | 3.46 |
|  | SBR/CMC | 13.9 | 2.61 |
|  | AmLA | 21.3 | 6.14 |
| C1 | PVDF | 9.1 | 1.12 |
|  | SBR/CMC | 10.2 | 1.41 |
|  | AmLA | 11.3 | 1.73 |
| C2 | PVDF | 17.3 | 4.05 |
|  | SBR/CMC | 24.7 | 8.25 |
|  | AmLA | 27.9 | 10.53 |

^a^ Calculated using the Randles–Sevcik equation:

$$i{}_{p}=2.69 \times{10}^{5} n^{3/2} AC_{Li^{+}} \sqrt{D_{{Li}^{+}}v}$$

where n = 2, A = 1.13 cm^2^, and C_Li+_ = 1.0 × 10^−3^ mol cm^−3^. The slopes are reported in units of mA (mV s^−1^)^−0.5^, and the corresponding unit factors were included in the D_Li+_ calculation.
